# Sex determination, longevity, and the birth and death of reptilian species

**DOI:** 10.1002/ece3.2277

**Published:** 2016-06-28

**Authors:** Niv Sabath, Yuval Itescu, Anat Feldman, Shai Meiri, Itay Mayrose, Nicole Valenzuela

**Affiliations:** ^1^ Department of Molecular Biology and Ecology of PlantsTel Aviv UniversityTel Aviv69978Israel; ^2^ Department of ZoologyTel Aviv UniversityTel Aviv69978Israel; ^3^ Department of Ecology, Evolution and Organismal BiologyIowa State UniversityAmesIowa50011

**Keywords:** Evolution and natural selection, life span and longevity, life history, phenotypic plasticity, sex chromosomes, sexual development, speciation, extinction, and net diversification, temperature‐dependent (TSD) and genotypic (GSD) sex determination, turtles, lizards, snakes, and squamate reptiles, vertebrate speciation and extinction

## Abstract

Vertebrate sex‐determining mechanisms (SDMs) are triggered by the genotype (GSD), by temperature (TSD), or occasionally, by both. The causes and consequences of SDM diversity remain enigmatic. Theory predicts SDM effects on species diversification, and life‐span effects on SDM evolutionary turnover. Yet, evidence is conflicting in clades with labile SDMs, such as reptiles. Here, we investigate whether SDM is associated with diversification in turtles and lizards, and whether alterative factors, such as lifespan's effect on transition rates, could explain the relative prevalence of SDMs in turtles and lizards (including and excluding snakes). We assembled a comprehensive dataset of SDM states for squamates and turtles and leveraged large phylogenies for these two groups. We found no evidence that SDMs affect turtle, squamate, or lizard diversification. However, SDM transition rates differ between groups. In lizards TSD‐to‐GSD surpass GSD‐to‐TSD transitions, explaining the predominance of GSD lizards in nature. SDM transitions are fewer in turtles and the rates are similar to each other (TSD‐to‐GSD equals GSD‐to‐TSD), which, coupled with TSD ancestry, could explain TSD's predominance in turtles. These contrasting patterns can be explained by differences in life history. Namely, our data support the notion that in general, shorter lizard lifespan renders TSD detrimental favoring GSD evolution in squamates, whereas turtle longevity permits TSD retention. Thus, based on the macro‐evolutionary evidence we uncovered, we hypothesize that turtles and lizards followed different evolutionary trajectories with respect to SDM, likely mediated by differences in lifespan. Combined, our findings revealed a complex evolutionary interplay between SDMs and life histories that warrants further research that should make use of expanded datasets on unexamined taxa to enable more conclusive analyses.

## Introduction

Vertebrate sex determination, or the commitment to a male or female developmental fate, can be triggered by an individual's genotype (genotypic sex determination [GSD]) or by environmental factors such as temperature (temperature‐dependent sex determination [TSD]; Bull 1983; Valenzuela and Lance 2004). GSD is found in all mammals, birds, and amphibians, while TSD exists in some fishes and in many reptiles (Bachtrog et al. 2014). Fewer examples of species with mixed mechanisms where GSD systems are overridden by certain temperatures have been documented in reptiles and fish (e.g., Shine et al. 2002; Yamamoto et al. 2014; Holleley et al. 2015). A clear explanation for the evolution of this diversity in sex‐determining mechanisms (SDM) remains elusive as our understanding of the causes and consequences of SDM turnover is inadequate.

Links between the evolution of SDMs and some important traits have been documented. For instance, transitions between SDM correlate with profound evolutionary changes, such as turtle genome reorganization (Valenzuela and Adams 2011), the evolution of viviparity in some marine reptiles (Organ et al. 2009), and adult sex ratio and demography of populations (Pipoly et al. 2015). Life history can also play a role in SDM turnover as theory predicts that lifespan influences whether TSD and GSD is adaptive, maladaptive, or neutral (Bull and Bulmer 1989; Valenzuela 2004a; Schwanz and Proulx 2008; Freedberg and Debenport 2014) and thus the tendency for TSD to be retained or replaced by GSD over evolutionary time. However, large‐scale empirical tests of these predictions are lacking.

Sex determination is expected to influence species diversification because it affects life history parameters (e.g., sexual development) and sex ratios and, consequently, population growth (via the effect of sex ratio on effective population size) that are linked to speciation and extinction (Bessa‐Gomes et al. 2004; Girondot et al. 2004; Valenzuela and Lance 2004; Bachtrog et al. 2014). Furthermore, sex determination strongly affects speciation in species with sex chromosomes as sex chromosomes often show the first signs of reproductive incompatibilities (Haldane 1922; Presgraves 2008; Elgvin et al. 2011). Yet, in groups where sex determination is evolutionarily labile, such as reptiles, evidence that sex determination is associated with diversification remains inconclusive as reports linking SDM and reptile speciation or extinction are conflicting. For instance, because TSD taxa are vulnerable to climate change, sex ratios could be skewed leading to extinction (Janzen 1994; Neuwald and Valenzuela 2011). TSD may thus lower diversification (i.e., speciation minus extinction) rates. However, TSD families appeared to have suffered lower extinction rates than GSD lineages during the climate change of the Cretaceous/Palaeogene transition (Silber et al. 2011; Escobedo‐Galvan and Gonzalez‐Salazar 2012). This observation suggests that TSD taxa may have been better adapted to past climate change than GSD taxa due to their phenotypic plasticity (Kallimanis 2009; Escobedo‐Galvan et al. 2011; Valenzuela et al. 2013). Conversely, transitions to GSD may be an adaptation to climate change to counter highly biased sex ratios in TSD taxa (Valenzuela and Adams 2011). Similarly, the transition to GSD was proposed to explain the adaptive radiation of extinct marine reptiles (Organ et al. 2009). Yet again, family‐level analyses found no relationship between diversification rates and the prevalence of GSD in Sauropsida (reptiles plus birds; Organ and Janes 2008). Thus, whether SDM is a causal driver or whether other correlated factors such as lifespan might be more important for diversification remains obscure.

Here, we take a phylogenetic, species‐level approach to examine the factors that influence the relative prevalence of SDMs in turtles and lizards. These two groups are ideal to address this question as TSD and GSD evolved multiple times independently within both groups (Valenzuela and Lance 2004). TSD is more common in turtles (78%) and GSD in lizards (86%) (Pokorná and Kratochvíl 2009; Valenzuela and Adams 2011). We test whether sex determination is associated with diversification rates, and whether alterative factors, such as lifespan's effect on transition rates, could explain the relative prevalence of SDMs in turtles and lizards. Based on existing data and analytical methods, we generate hypotheses to guide future research.

## Methods

### Data and phylogenies

An initial reptilian SDM database was obtained from (The Tree of Sex Consortium, 2014) and complemented with an extensive literature search (Ota et al. 1992; Gamble 2010; Pokorná et al. 2011, 2014; Trifonov et al. 2011; Badenhorst et al. 2013; Matsubara et al. 2013, 2014; Gamble et al. 2014, 2015; Koubová et al. 2014; Pokorna et al. 2014; Rovatsos et al. 2014a,b; Schmid et al. 2014; Sulandari et al. 2014). The resulting dataset contains information for 87 turtle and 303 lizard species (Table S1a) that have been studied across families (Table S1b). TSD and GSD were defined following (Valenzuela et al. 2003). To account for species that possess mixed sex‐determining mechanism where GSD and TSD coexist (termed “GSD+EE” by Valenzuela et al. 2003), and for species for which the evidence for TSD is weak (Harlow 2004; Valenzuela 2004b; Table S1a), we ran alternative analyses using one or the other SDM classification to test the robustness of our results. We used a dated phylogeny of 314 turtle species (Valenzuela and Adams 2011; ~96% of all estimated species by van Dijk et al. 2014), and one of 2899 lizard species (Pyron and Burbrink 2014) [~47% of estimated 6176 species (Uetz and Hosek 2015) representing all recognized families and subfamilies]. Of these, all 87 turtle species in the SDM dataset were present in the turtle phylogeny, and 279 lizard species with known SDM were represented in the phylogeny. Snakes, which share a single ZZ/ZW GSD system (Rovatsos et al. 2015), are nested within lizards. We thus analyzed an additional squamate dataset that included both lizards and snakes. Results did not differ from those found in lizards alone. Unless noted otherwise, because most of the methods that we used (see details below) do not account for unknown character states, the trees were pruned to include only taxa with known SDM.

### Diversification analyses

Three different approaches, ranging from nonparametric, semiparametric, and fully parametric, were applied to test for SDM effect on lineages diversification rates. To assess statistical significance, each analysis was complemented with a parametric bootstrap approach to obtain the null distribution expected under a scenario depicting those of the empirical datasets.

First, we used the MacroCAIC method (Agapow and Isaac 2002) implemented in R package “caper” (Orme et al., 2013), to test for a correlation between SDM and species richness under the phylogenetically independent contrast paradigm (Felsenstein 1985). The method produces contrasts across a clade, including only contrasts with a minimal number of species (MNS), and linear regressions are then fitted to these contrasts (e.g., using MNS = 20, the mean size and richness value of all contrasts in the clade that have at least 20 species are calculated). We thus applied the method to turtles and lizards using MNS cutoffs of 10, 20, 30, and 40.

To control for potentially inflated false‐positive rate of MacroCAIC (Freckleton et al. 2008), we used a parametric bootstrapping approach to obtain the null distribution of the *F*‐statistic inferred by MacroCAIC expected for a neutral character with no effect on diversification patterns. Specifically, we simulated 1000 random distributions of neutral characters (assuming no effect on diversification) on the same empirically derived phylogenies of turtles and lizards. To obtain the simulated parameter values, we first estimated the two transition rates (GSD to TSD and TSD to GSD) according to a MK2 model (using make.mk2 function within the package *diversitree* [FitzJohn 2012]) and the root state set to TSD (which was inferred as the root state, see Results). We then simulated a binary trait along the tree according to the inferred transition rates (using sim.character function within the package *diversitree* [FitzJohn 2012]), starting, again, with the root state set to TSD. We then applied MacroCAIC on each simulated set and recorded the *F*‐statistic values. Finally, the empirically derived *F*‐statistic value was compared to the corresponding simulated distributions to obtain a *P* value according to the proportion of simulated values that are equal or greater than the observed value.

Second, we used STRAPP (“STructured RAte Permutations on Phylogenies”), a recently developed semiparametric test for detecting trait‐dependent diversification (Rabosky and Huang 2015). STRAPP first divides the input phylogeny into distinct diversification regimes, without considering the analyzed trait, as estimated by BAMM (Rabosky et al. 2013). It then treats these regimes as distinct data points to test for a trait effect on diversification. Unlike BiSSE (Maddison et al. 2007), which was recently shown to exhibit an elevated Type I error rate in the estimation of diversification rates (FitzJohn 2012; Rabosky and Goldberg 2015), STRAPP does not reconstruct character changes and diversification simultaneously. Consequently, STRAPP was shown to have low Type I error rates that are robust to combinations of character state frequencies and evolutionary rates, as well as to missing data, such that it is proposed for use even when character state data are available for a small fraction of the species in a phylogeny (Rabosky and Huang 2015). We note, however, that the improved lower rate of Type I errors in STRAPP is possibly accompanied by reduced sensitivity (Rabosky and Huang 2015). Thus, results using STRAPP would be conservative. We ran BAMM on each phylogeny, for 2,000,000 generations, keeping event data every 1000 steps. We then removed the first 10% steps as burn‐in and applied STRAPP using 10,000 permutations.

Finally, we conducted a third analysis of diversification as a function of SDM using the BiSSE modeling framework (Maddison et al. 2007; Data S1) and alleviated the potential problem of false‐positives with extensive simulations to test the robustness of the results following (FitzJohn 2012; Rabosky and Goldberg 2015). As detailed above, we simulated random distributions of neutral characters that have no effect on diversification on the same phylogenies and then tested whether the log‐likelihood difference for the competing models is more extreme for the real datasets than what could be expected by chance for neutral simulated traits (Rabosky and Goldberg 2015). For the BiSSE analyses, the full phylogenies containing 314 turtles, 2899 lizards, and 4161 squamates were used, but we also provide the results obtained using the pruned trees (Data S1).

### Transition rates analyses

We tested whether the transition rate from GSD to TSD (*q*_GT_) is different than from TSD to GSD (*q*_TG_), using the MK2 model within the R package *diversitree* (FitzJohn 2012). We used maximum‐likelihood (ML) analysis to compare two nested models, one in which *q*_GT_ is different from *q*_TG_, and one where the rates are equal. The LRT was then used to choose the best‐fit model.

As a second method, we tested for differences in transitions rates using a Markov chain Monte Carlo (MCMC) sampling approach (FitzJohn et al. 2009) to estimate the posterior probability distributions of the two transition rate parameters. We used an exponential prior distribution with mean set to 0.1. MCMC chains were run for 2000 steps with the first 25% discarded as burn‐in. To test whether transition rates differ between SDMs, we calculated the proportion of MCMC steps (i.e., the posterior probability, PP) in which *q*_TG_ was higher than *q*_GT_. PP value above 0.975 or below 0.025 indicates a significant difference between the two rates. To examine whether the estimation of transition rates is affected by accounting for trait‐dependent diversification, we applied the BiSSE framework (Maddison et al. 2007) as implemented in *diversitree* version 0.9.7 (FitzJohn 2012). We used the “skeletal” tree approach (FitzJohn et al. 2009), which accounts for the sampling fraction of species in the phylogeny out of the total number of species in the clade (assuming an equal sampling fraction for both TSD and GSD). This method was used to estimate the speciation rates of lineages in states GSD and TSD and extinction rates, in addition to the transition rate parameters. Similar to the ML analysis of the MK2 model, we compared two nested models, one in which *q*_GT_ is different from *q*_TG_, and one where the rates are equal (while speciation and extinction rates are constrained to be equal), using LRT. We note that BiSSE inference could be biased due to several characteristics of our data, including small sample size (turtles), high tip ratio bias (overprevalence of one observed character state), and incomplete sampling (lizards; Davis et al. 2013). Parametric bootstrap was used to test the false‐positive rate of each approach; namely, we simulated character states using the sim.character function (R package *diversitree* [FitzJohn 2012]), with the root state set to TSD. Here, we applied an equal transition rate model. The rate parameters used in the simulations were identical to those estimated from the real data using the constrained MK2 model. We then compared the empirically derived statistics (∆LL in the ML and PP in the MCMC analysis) to the corresponding simulated distributions to obtain a *P* value according to the proportion of simulated values that are equal or greater than the observed value.

We also tested the effect of missing data on the estimation of the transition rates in BiSSE, which could afflict the lizard dataset more strongly. For this, we simulated random trees with 1000 tips with equal speciation rates (*λ* = 0.1), no extinction, and varying transition rates (q01 = 0.1, q10 = 0.1, 0.05, 0.025) and carried out 100 simulations for each parameter combination (Data S1). In each simulation, the data were analyzed by BiSSE with 100, 25, or 5% of the state data while the rest were converted to missing data (Fig. S3).

### Ancestral state reconstruction

We used a Markov model of trait change (the MK2 model within the R package *diversitree* (FitzJohn 2012)) to reconstruct the ancestral state at the root with the asr.marginal function within the R package *diversitree* (FitzJohn 2012). Because diversification could bias the inference of ancestral state reconstruction (King and Lee 2015), we also reconstructed the ancestral state using a BiSSE model (again, with the asr.marginal function), assuming equal speciation and extinction rates of GSD and TSD states (as the alternative model of unequal diversification rates was not supported; see Results).

### Estimating shifts in lifespan in association with SDM

We examined the possible correlation of lifespan with SDM evolution using OUwie (Beaulieu et al. 2012). Lifespan data were obtained from (Tacutu et al. 2013; Scharf et al. 2015) and were log‐transformed. First, we assessed whether the rate of lifespan evolution differs between TSD and GSD lineages by comparing the fit of single‐ and two‐rate models of Brownian motion (BM) evolution. The best‐fit model was chosen using the LRT. The two‐parameter model requires partitioning of the tree into distinct regimes (i.e., a reconstructed phylogenetic history of GSD and TSD lineages, which was performed again with the asr.marginal function). Second, we used OUwie to test whether TSD and GSD lineages differ in their evolutionary trajectory (optimum value) for lifespan. Two nested models were compared using the LRT. In the first (OU1), there is a common optimum for GSD and TSD lineages while the second model (OUM) allows each SDM state to have a distinct optimum. We used AIC to compare the four models (BM1, BM2, OU1, and OUM).

## Results

### Diversification analyses

We explored whether differential diversification explains the contrasting abundance of SDMs in turtles and lizards in order to illuminate the causes and consequences of SDM evolution in these two lineages. Results were robust to the inclusion of snakes along with lizards in a squamate dataset during analyses, as they did not differ from the results obtained with lizards alone.

In turtles, standard MAcroCAIC procedures using the *F*‐statistic predicted a marginal statistical support for higher species richness in TSD clades than in GSD clades (*P* = 0.046, in MNS = 30 and 40; *P* > 0.25 for MNS = 10 and 20; Table 1). However, results from the parametric bootstrap showed that the observed differences are not significantly different than what was expected by chance in any group (*P* > 0.12, Table 1). In lizards and squamates, no significant difference between SDMs was observed under both statistical measures (*P* > 0.5 in all MNS values, Table 1). BAMM predicted 1, 7, and 16 rate shifts in diversification for turtles, lizards, and squamates, respectively (Table S3). The STRAPP method (Rabosky and Huang 2015) consistently detected no significant association between diversification rate estimates and SDM (*P* > 0.5 in all cases) (Table 2). Results were robust to using alternative SDM assignment for species with mixed or equivocal SDM (Tables 2 and S2).

**Table 1:** MacroCAIC results

Group	MNS^a^	*r* ^2^	Slope	*P* value	Simulation *P* value
Turtles	10	−0.05	3.56	0.717	0.759
	20	0.06	33.30	0.276	0.316
	30	0.71	66.90	0.046	0.148
	40	0.71	66.90	0.046	0.135
Lizards	10	−0.01	−2.79	0.734	0.699
	20	−0.02	−4.79	0.819	0.808
	30	−0.03	−3.48	0.879	0.875
	40	−0.04	−1.05	0.971	0.971
Squamates	10	−0.01	−2.43	0.75	0.74
	20	−0.02	−5.08	0.80	0.86
	30	−0.02	−3.84	0.86	0.85
	40	−0.03	−5.98	0.80	0.83

a MNS, minimal number of species included for computing contrasts.

**Table 2:** STRAPP results

Group	*P*	*P* (alternative SDM assignment)
Turtles	0.95	0.96
Lizards	0.67	0.73
Squamates	0.56	0.65

Consistent with these results, although initial analysis using the BiSSE approach (Maddison et al. 2007) identified differences by SDM in diversification rates in turtles, lizards, and squamates (Data S1), the results from our parametric bootstrapping procedure using neutral binary traits showed that, in all groups, the inferred differences using BiSSE are not significantly different than expected by chance (Data S1).

### Transition rates analyses

Transitions from TSD to GSD were significantly more frequent than transitions from GSD to TSD in lizards and squamates, while in turtles transition rates were not significantly different, regardless of the analysis used (ML or Bayesian; Table 3). Unlike the diversification analyses, here the null model was rejected also when applying the parametric bootstrap approach (Table 3). Results were robust to accounting for diversification (using a BiSSE model) and to using alternative SDM assignment for species with mixed or equivocal SDM (Table S3). Our simulations also indicated that while greater sampling diminishes the variance in the transition rate estimates, the estimates are generally unbiased with the average transition rate estimate centered around to true value also when a large proportion of the tips do not contain trait data (Fig. S3).

**Table 3:** Summary of transition rate parameters estimates using the MK2 model with both maximum‐likelihood and Bayesian (MCMC) methodologies and BiSSE for the turtles, lizards, and squamate datasets

Group	Analysis	q_GT_	q_TG_	Significance^a^	Simulation *P* value
Turtles	Maximum likelihood	8.6 × 10^−07^	0.0017	0.10	0.11
	MCMC	0.0016	0.0021	0.73	0.09
	BiSSE	6.5 × 10^−06^	0.0018	0.10	0.14
Lizards	Maximum likelihood	5.7 × 10^−04^	0.0119	**2.6 × 10** ^**−05**^	**<0.001**
	MCMC	9.5 × 10^−04^	0.0120	**1**	**<0.001**
	BiSSE	5.9 × 10^−04^	0.0119	2.5 × 10^−05^	**<0.001**
Squamates	Maximum likelihood	3.0 × 10^−04^	0.0121	**2.7 × 10** ^**−07**^	**<0.001**
	MCMC	5.0 × 10^−04^	0.0124	**1**	**<0.001**
	BiSSE	3.0 × 10^−04^	0.0122	2.7 × 10^−07^	**<0.001**

a Significance is estimated with likelihood ratio test for the maximum‐likelihood and BiSSE analyses; Significance of the MCMC analyses is estimated by calculating the proportion of MCMC steps (i.e., the posterior probability, PP) in which *q*_TG_ was higher than *q*_GT_. PP value above 0.975 or below 0.025 indicates a significant difference between the two rates. Significant *p*‐values are denoted in bold.

### Ancestral state reconstruction

**Figure 1:**
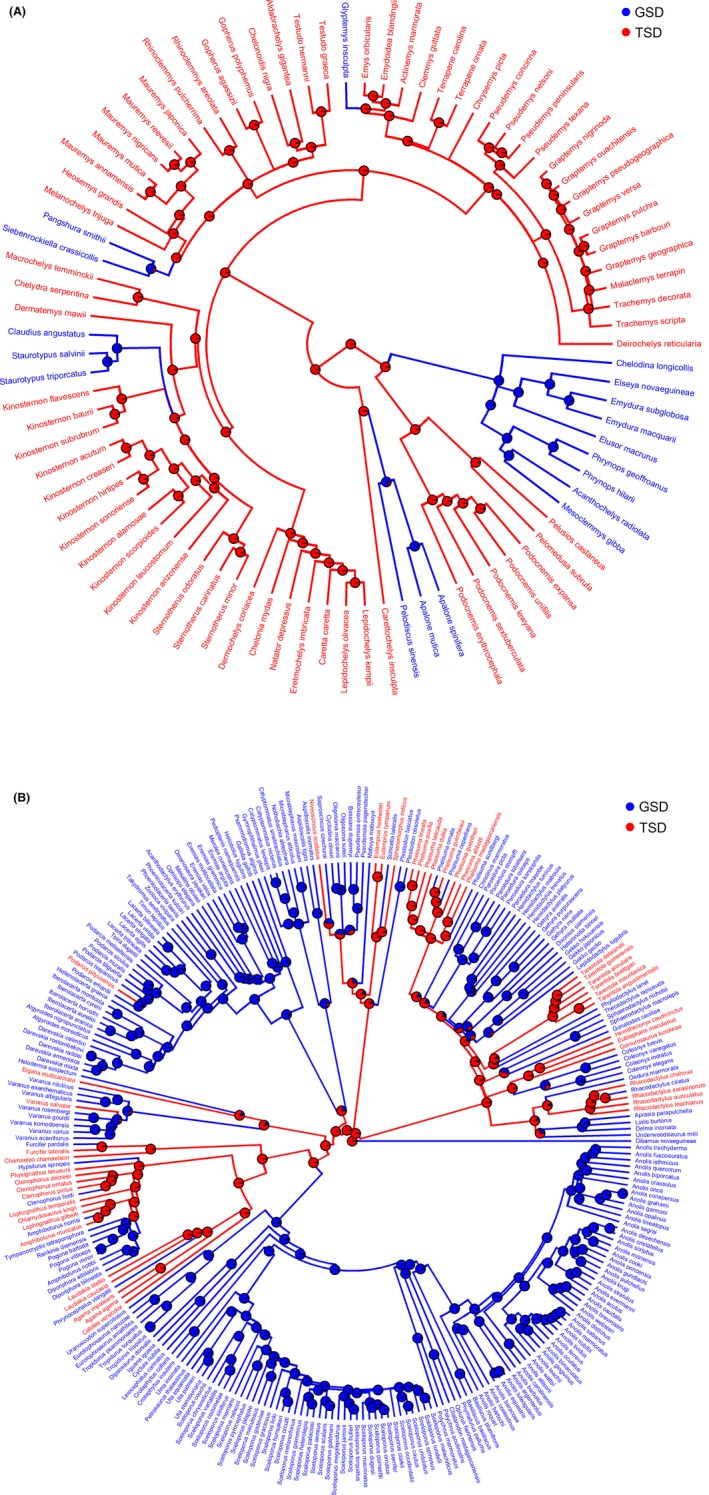
ML ancestral reconstruction of sex‐determining mechanisms in (A) turtles and (B) lizards. Pie charts denote the state probabilities at ancestral nodes.

Ancestral state reconstruction revealed that TSD is ancestral for both turtles and lizards (Fig. 1). Results were robust to the inclusion of snakes in the analyses (Fig. S1). The inference of ancestral TSD state was not affected when BiSSE was applied to account for species diversification or when using the alternative SDM classification for taxa with either mixed SDM or with weakly supported TSD (Fig. S1). Our results agree with previous reconstructions obtained with smaller datasets using ML in turtles (Valenzuela and Adams 2011) and maximum parsimony in squamates (Pokorná and Kratochvíl 2009). Altogether, our results suggests that the ancestral TSD state in both clades, combined with the asymmetry in lizard (but not turtle) transition rates (TSD‐to‐GSD surpass GSD‐to‐TSD), explains the observed prevalence of TSD turtles (via TSD retention) and GSD lizards (via TSD‐to‐GSD transitions) observed in nature.

### Coevolution of SDM and longevity

**Figure 2:**
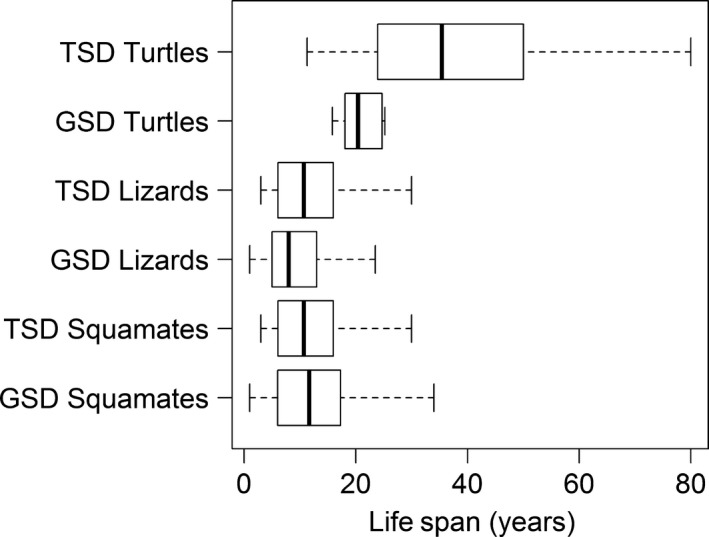
Box plots depicting longevity values for TSD and GSD turtles, lizards, and squamates. A significant difference between TSD and GSD lineages was detected in turtle longevity using the phylogenetic Ornstein–Uhlenbeck model (see text for details).

To examine the possibility that differences in longevity might have influenced the evolutionary patterns of lineages in both groups, we modeled the evolution of lifespan with respect to SDM. The BM analysis showed that TSD turtles underwent greater lifespan evolution than their GSD counterparts, whereas the opposite was true in lizards where GSD lineages experienced greater lifespan evolution than TSD lizards (no differences were detected in squamates). In turtles, this greater evolutionary rate resulted in contrasting lifespan optima by SDM, whereas no differences were detected in lizards or squamates. Namely, we found that TSD turtles evolved toward greater longevity than GSD turtles (35.9 and 22.6 years, respectively; *P* = 0.018), whereas in lizards (and squamates), lifespan did not differ significantly between SDMs (Table 4; Fig. 2). When we compared the four models (BM1, BM2, OU1, and OUM) together, we found that in all datasets the OU models fit the data significantly better. Results were robust to using alternative SDM assignment for species with mixed or equivocal SDM (Table S3).

**Table 4:** Log‐likelihood differences (∆LL) obtained between the single (BM1)‐ and two (BM2)‐rate Brownian motion models of evolution, and between the single (OU1) and two (OU2) optimums, as estimated for lifespan in turtles, lizards, and squamates. $\sigma_{\mathrm{GSD}}^{2}$, $\sigma_{\mathrm{TSD}}^{2}$, optimum_GSD_, and optimum_TSD_: estimated parameters for GSD and TSD lineages. Significant *p*‐values are denoted in bold

Group	LogLiks BM1	LogLiks BM2	BM *P*‐value^a^	$\sigma_{\mathrm{GSD}}^{2}$	$\sigma_{\mathrm{TSD}}^{2}$	LogLiks OU1	LogLiks OU2	OU *P*‐value^b^	Optimum_GSD_	Optimum_TSD_
Turtles	−90.7	−84.7	**0.0005**	0.361	2.8368	−72.9	−70.1	**0.0181**	22.6204	35.8875
Lizards	−192.9	−189.9	**0.0135**	3.3016	1.6455	−163.8	−162.8	0.1496	7.9104	10.0869
Squamates	−246.4	−245.2	0.1126	2.5472	1.6852	−220.7	−220.7	0.7911	9.5098	10.019

a *P*‐value comparing the fit of a single‐ and two‐rate BM models based on the likelihood ratio test.

b *P*‐value comparing the fit of a single and two OU models based on the likelihood ratio test.

## Discussion

### SDM and diversification

Here, we examined the association of sex determination and species richness in reptiles by examining possible differences in diversification rates between TSD and GSD lineages, together with the transition rates between SDM states, in the two reptilian groups with labile sex determination – turtles and lizards (with and without the inclusion of snakes). We did not detect a significant influence of SDMs on turtle and lizard diversification using three alternative methods. Our findings were not affected by the inclusion of snakes in the analyses, nor by the few taxa with documented mixed SDM or by those with equivocal SDM assignment. Instead, the inferred transition rates between SDMs (which differed between the groups examined), coupled with TSD ancestry, could explain the predominance of GSD in lizards and TSD in turtles, without a significant difference between diversification rates.

This result is surprising because SDMs affect demographic and reproductive traits, and consequently, it is expected that SDM should influence species diversification and the prevalence of TSD or GSD found in nature. Indeed, SDMs affects population sex ratios, which in turn affect the effective population size, and, ultimately, population growth and the rate of loss of genetic variation (Hartl and Clark 1989) – all of which are factors underlying adaptation, speciation rates, and extinction probabilities (Bessa‐Gomes et al. 2004; Girondot et al. 2004). Our findings agree with previous family‐level analysis of reptiles and birds, which detected no association between speciation rates and SDMs (Organ and Janes 2008), although it should be noted that their results could also be due to the lower power of family‐level analyses (Organ and Janes 2008) and the fact that their study combined a family‐level tree with a model that assumes complete sampling (Organ and Janes 2008). The lack of evidence of an effect of SDM on diversification contradicts the expectation that TSD species should be more vulnerable to extinction as climate change can drastically bias TSD sex ratios (Janzen 1994; Neuwald and Valenzuela 2011), as well as counter reports that TSD reptilian families suffered lower extinction rates than GSD families during the Cretaceous/Palaeogene transition (Silber et al. 2011; Escobedo‐Galvan and Gonzalez‐Salazar 2012).

The observed lack of support for a relationship between SDM and diversification could also be due to the sparsity of the data (Table S1b) or the limitations of the methods (Freckleton et al. 2008; Maddison and FitzJohn 2015; Rabosky and Goldberg 2015). However, it should be noted that the same independence between SDM and diversification was detected here with three alternative methods (MacroCAIC, STRAPP, and the permutation analyses to test BiSSE results). Yet, all methods employed intrinsically assume a homogenous evolutionary process for both transition and diversification rates. That is, the model is time homogenous and similar across different clades of the phylogeny. This assumption is rather questionable for the large clades analyzed here. However, the sparsity of the data did not allow us to explore more sophisticated models that require a larger number of parameters. Taken together, while our results indicate that asymmetries in transition rates rather than diversification rates lead to the differential SDM diversity observed in squamates, the jury awaits for improved and well‐vetted analytical methods plus the collection of additional information on sex determination in reptiles. Fortunately, SDM data are growing at an accelerated pace thanks to the use of a variety of molecular techniques to complement classic incubation experiments (Ota et al. 1992; Gamble 2010; Pokorná et al. 2011, 2014; Trifonov et al. 2011; Badenhorst et al. 2013; Matsubara et al. 2013, 2014; Mu et al. 2013; Gamble et al. 2014, 2015; Koubová et al. 2014; Pokorna et al. 2014; Rovatsos et al. 2014a,b; Schmid et al. 2014; Sulandari et al. 2014; Valenzuela et al. 2014; Montiel et al. 2016). Extensive research over 50 years has uncovered SDM information in all turtle families (except Platysternidae) for at least 1 species per family, whereas 9 of 37 lizard families remain unstudied. The coverage varies across families, from <10–68% for turtle families that are not monotypic, compared to 1–50% in lizards (Table S1b). Notably, the existing data and methods permit some important insights into why GSD is more prevalent than TSD in squamates while TSD turtles abound over GSD turtles, and these working hypotheses should foster even further research in this area.

### SDM and species richness

The contrasting relative prevalence of TSD and GSD in turtles and lizards (as well as squamates) can be explained by their differences in the transition rates between SDMs; namely, in turtles, transition rates between TSD and GSD were similar, such that the higher abundance of TSD turtles derives from the greater retention of the ancestral TSD condition. In contrast, lizards (including and excluding snakes) shifted from TSD into GSD much more often than from GSD to TSD, resulting in greater abundance of GSD lizards overall (and all snakes retain a ZZ/ZW GSD system that evolved at their split from lizards (Rovatsos et al. 2015). In general, a lack of difference between transition rates in turtles could be due to a relatively small number of transitions overall or to an overall short time lineages have been in the GSD state (i.e., “lower rate” vs. “lower opportunity” to transition).

### Longevity, sex determination, and diversification

**Figure 3:**
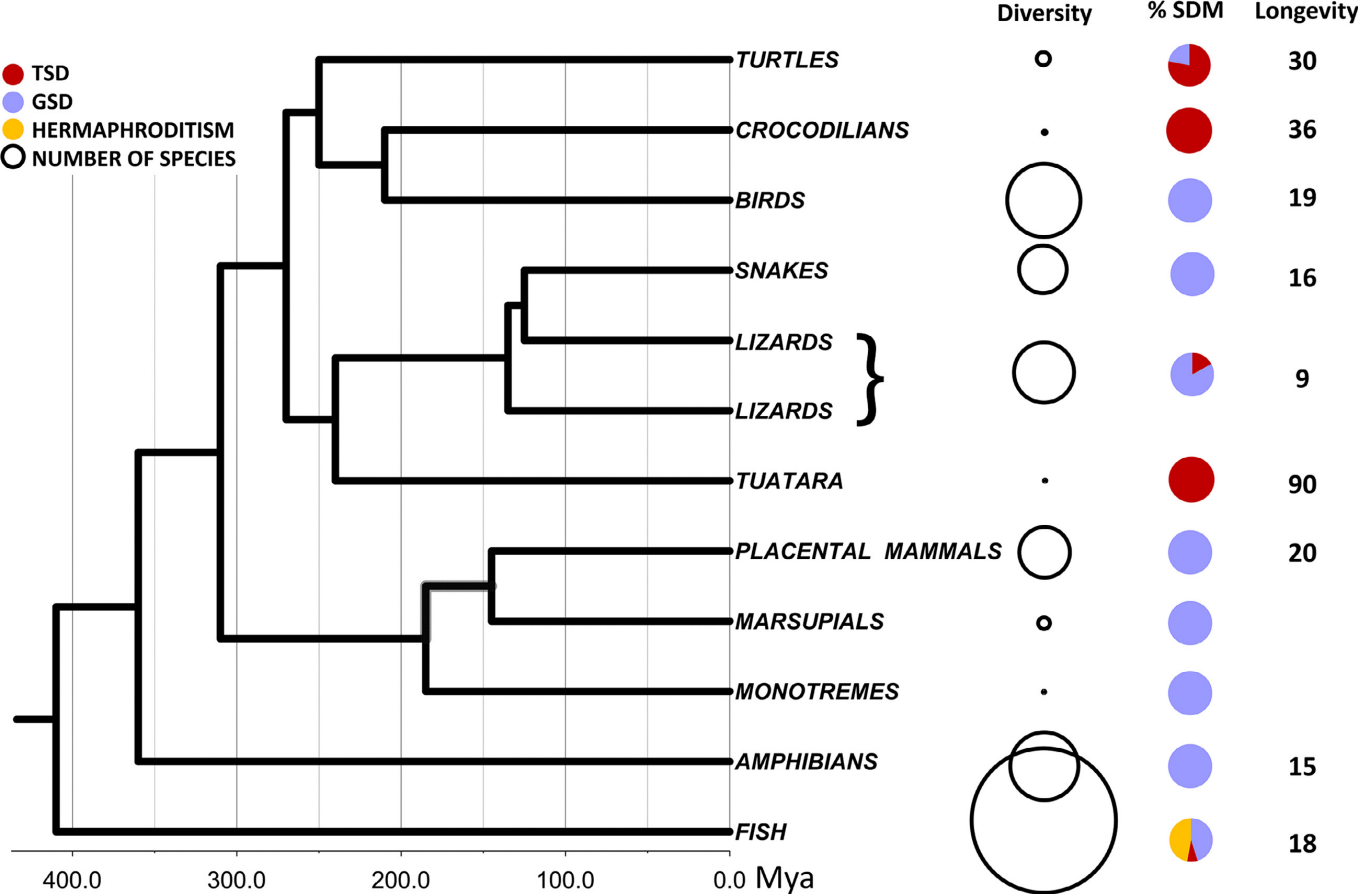
Species diversity, sex determination and longevity of extant vertebrates. Species numbers per lineage vary from 1 (tuatara) to >33,000 (fish) (Eschmeyer and Fong 2014; Frost 2014; Hay et al. 2010; Uetz and Hosek 2015; van Dijk et al. 2011). Sex determination from sources cited in the text. Divergence times as per Chiari et al. (2012) and Jones et al. (2013). Average longevity from (Tacutu et al. 2013) in years (Data S1). Open circle size is proportional to species number per clade. Values are presented for lizards overall, despite the paraphyly with snakes falling within the lizard clade.

Why did lizards give up TSD so readily while turtles thrived with TSD? We hypothesize that differences in individual longevity between these two clades are key, because lifespan influences whether TSD is adaptive, maladaptive, or neutral (Bull and Bulmer 1989; Valenzuela and Lance 2004). Turtles live, on average, over three times as long (~30 years) as lizards (~9 years) (Tacutu et al. 2013; Scharf et al. 2015; Fig. 3). This may even be an underestimate because lizard data include an overrepresentation of large species that tend to be longer‐lived (Tacutu et al. 2013). These differences in lifespan are relevant because TSD populations are vulnerable to producing drastically biased sex ratios in any given season, which may imperil population survival of short‐lived species, while longevity lessens this detrimental effect by providing reproductive assurance (Girondot and Pieau 1996; Valenzuela and Lance 2004; Grayson et al. 2014). Namely, in theory, a population of an annual species that produces a single sex during a drastically cold or warm year could go extinct in a single generation, while long‐lived TSD taxa would more effectively average sex ratios over multiple years (Girondot and Pieau 1996) because by the time individuals reach maturity, and during their multiple reproductive years, potential mates would have been recruited into the population. Additionally, TSD may lead to the accumulation of deleterious mutations particularly under shorter lifespan (because biased sex ratios produced by TSD reduce effective population sizes; Freedberg and Debenport 2014), and in combination with environmental fluctuation regimes (Schwanz and Proulx 2008). GSD would thus be expected to persist more frequently in short‐lived lineages, while TSD would be expected to persist more readily in longer‐lived lineages. Under such circumstances, heritable genetic variation underlying sexual development of TSD species (Sarre et al. 2011; Valenzuela et al. 2013) might enable the persistence and adaptation of long‐lived taxa during changing climatic conditions. This could explain the persistence of TSD turtles in ways that may have been precluded for many shorter‐lived lizards. The hypothesis that longevity mediates TSD retention was supported when we tested whether TSD and GSD lineages differ in their evolutionary trajectory for lifespan and found that lifespan of TSD turtles evolved toward greater values (and are consequently longer‐lived) than their GSD counterparts (lizards showed a similar tendency but these differences were not significant [Table 4]). Concordant with this notion, the other reptilian lineages that have only TSD are also long‐lived (Fig. 3), that is, crocodilians and the rhynchocephalian (tuatara). We note that if the pace of climate change is too rapid – as occurs today (Diffenbaugh and Field 2013) – adaptive responses such as those inferred here may be limited, particularly for the many TSD taxa that are already endangered and suffer from small population sizes and drastic habitat degradation (van Dijk et al. 2014).

Then, how could TSD evolve or persist in short‐lived taxa, such as some lizards and fish? TSD must be much more adaptive than GSD in short‐lived taxa to compensate for the costs associated with fluctuating sex ratios (e.g., sensu Charnov and Bull 1977). Under the Charnov–Bull model, TSD is adaptive and would be favored over GSD, if (1) the environment is patchy (in space or time) and unpredictable by the developing offspring or their parents, (2) the temperature (or a correlated variable) experienced during early development confers differential lifetime fitness to each sex, and (3) individuals mate at random among patches (Charnov and Bull 1977). This model was elegantly demonstrated to apply for *Amphibolurus muricatus* lizards (Warner and Shine 2008), and for *Menidia menidia* fish (Conover and Heins 1987). In both these short‐lived vertebrates, spring/summer temperature (when sexual development occurs) is positively correlated with the time available for growth before winter, which determines adult size. In both cases, female fitness (via fecundity gains) increases with body size more than in males (Conover and Heins 1987; Warner and Shine 2008). Females of both species develop at colder temperatures naturally experienced early in the reproductive season, grow for a longer time and attain larger adult sizes, while males develop at warmer temperatures experienced later in the year, experience a shorter growing season and, consequently, attain smaller adult sizes (Conover and Heins 1987; Warner and Shine 2008), in close accord with the Charnov–Bull model. Thus, given the right conditions, TSD can evolve or persist in shorter‐lived taxa. Similar advantages may also exist in TSD turtles (Shine 1999; Valenzuela and Lance 2004), and such adaptive significance would only reinforce the persistence of TSD in chelonians.

In summary, our data support the hypothesis that diversification was not affected by SDM and that the high transition rates from TSD to GSD in lizards accounts for the high abundance of GSD lineages in this group, while TSD prevalence in turtles seems to reflect the retention of the ancestral character state. We hypothesize that turtle longevity helps them cope with fluctuating sex ratios. In contrast, we propose that the general shorter lifespan of lizards hinder TSD persistence (except under strong selection) favoring the transitions to GSD and contributing to their overall prevalence we observe in nature. Thus, our results underscore that turtles and lizards appear to have followed different evolutionary trajectories with respect to SDM likely mediated by differences in life‐history traits. An urgent need remains to expand the existing SDM and life‐history information of the many reptiles that remained unstudied, so as to enable more conclusive analyses. Our work contributes to ongoing efforts to study phenotypic macroevolution in a comprehensive manner to illuminate the relative success and demise of distinct branches of the tree of life, their causes and consequences, and their potential to adapt to a changing world.

## Conflict of Interest

None declared.

## Supporting information


**Figure S1.** ML ancestral reconstruction of sex‐determining mechanisms in (A) squamates, and using the alternative SDM classification in (B) squamates and (C) lizards.
**Table S1A.** Dataset used in this study.
**Table S1B.** Taxonomic coverage of turtle and squamate families used in this study.
**Data S1.** Results using alternative SDM assignment for species with mixed or equivocal SDM as listed in Table S1:
**Table S2.** MacroCAIC results using alternative SDM assignment.
**Table S3.** BAMM estimation for the number of rate shifts in diversification. The number of rates shifts with the highest probability in each group is marked in bold.
**Table S4.** Summary of transition rate parameters estimates using the MK2 model with both Maximum Likelihood and Bayesian (MCMC) methodologies and BiSSE for the turtles, lizards, and squamate data sets using the alternative SDM assignment.
**Table S5.** Log likelihood differences (ΔLL) obtained between the single (BM1) and two rate (BM2) Brownian motion models of evolution, and between the single (OU1) and two (OU2) optimums, as estimated for lifepan in turtles, lizards and squamates. *σ*2GSD and *σ*2TSD, OptimumGSD, and OptimumTSD: estimated parameters for GSD and TSD lineages using the alternative SDM assignment.
**Data S2.** BiSSE Analyses.
