# Sex determination, longevity, and the birth and death of reptilian species

**DOI:** 10.1002/ece3.2559

**Published:** 2016-11-01

**Authors:** Niv Sabath, Yuval Itescu, Anat Feldman, Shai Meiri, Itay Mayrose, Nicole Valenzuela

## Coevolution of SDM and Longevity

Some errors were detected in the turtle longevity values reported in the Supplemental Data Table S1a (the corrected dataset can be found below). This mistaken dataset was used in the longevity analyses reported in the paper, such that some results and conclusions need to be corrected as follows.

1. After re‐running the analyses using the corrected dataset, we found that the trend for greater lifespan evolution observed in TSD turtles compared to GSD turtles using the BM analysis is only statistically marginally significant (*p* = .087 instead of the reported *p* = .0005). Likewise, the trend of TSD turtles to evolve toward a higher lifespan optima than GSD turtles (36.0 and 19.6 years, respectively) is again marginal (*p* = .059 instead of the reported *p* = .018) (see Corrected Table 4 below). However, the direction of the trends themselves remains unchanged, namely the values of lifespan evolution and lifespan optimum are larger for TSD turtles than GSD turtles. These new results were robust to using alternative SDM assignment for species with mixed or equivocal SDM.
2. Consequently, the support for the hypothesis that longevity mediates TSD retention is qualitative (i.e., TSD turtles evolved toward greater values than GSD turtles) and not quantitative as reported in the manuscript (i.e., the trend of TSD turtles evolving toward greater values than GSD turtles is only marginally significant).

The authors regret this error.

**Corrected Table 4:** Log‐likelihood differences (∆LL) obtained between the single‐ (BM1) and two‐rate (BM2) Brownian motion models of evolution, and between the single (OU1) and two (OU2) optimums, as estimated for lifespan in turtles, lizards, and squamates. σ^2^_GSD_ and σ^2^_TSD_, optimum_GSD_, and optimum_TSD_: estimated parameters for GSD and TSD lineages

**Table:** 

Group	LogLiks BM1	LogLiks BM2	BM*p*‐value^a^	σ^2^ GSD	σ^2^ TSD	LogLiks OU1	LogLiks OU2	OU*p*‐value^b^	Optimum GSD	Optimum TSD
Turtles	−63.5	−62.0	.087	0.57	1.52	−59.4	−57.6	.059	19.6	36.0
Lizards	−192.9	−189.9	**.013**	3.30	1.65	−163.8	−162.8	.150	7.9	10.1
Squamates	−246.4	−245.2	.112	2.55	1.69	−220.7	−220.7	.791	9.5	10.0
