# Supplementary material for: Sex determination, longevity, and the birth and death of reptilian species
Source: Ecol Evol. 2016 Jun 28;6(15):5207–20. doi: 10.1002/ece3.2277 (PMC4984498; doi:10.1002/ece3.2277)
Supplement: Supplementary file 1 — Figure S1. ML ancestral reconstruction of sex‐determining mechanisms in (A) squamates, and using the alternative SDM classification in (B) squamates and (C) lizards. Table S1A. Dataset used in this study. Table S1B. Taxonomic coverage of turtle and squamate families used in this study. Data S1. Results using alternative SDM assignment for species with mixed or equivocal SDM as listed in Table S1: Table S2. MacroCAIC results using alternative SDM assignment. Table S3. BAMM estimation for the number of rate shifts in diversification. The number of rates shifts with the highest probability in each group is marked in bold. Table S4. Summary of transition rate parameters estimates using the MK2 model with both Maximum Likelihood and Bayesian (MCMC) methodologies and BiSSE for the turtles, lizards, and squamate data sets using the alternative SDM assignment. Table S5. Log likelihood differences (ΔLL) obtained between the single (BM1) and two rate (BM2) Brownian motion models of evolution, and between the single (OU1) and two (OU2) optimums, as estimated for lifepan in turtles, lizards and squamates. σ2GSD and σ2TSD, OptimumGSD, and OptimumTSD: estimated parameters for GSD and TSD lineages using the alternative SDM assignment. Data S2. BiSSE Analyses. [file ECE3-6-5207-s001.pdf]

SUPPORTING INFORMATION
Sex Determination, longevity, and the Birth and Death of Reptilian Species

Niv Sabath, Yuval Itescu, Anat Feldman, Shai Meiri, Itay Mayrose, Nicole Valenzuela

1. **Table S1a:** **Dataset used in this study [taxonomy follows (Uetz & Hosek, 2015)]**
   Table S1b: **Taxonomic coverage of turtle and squamate families used in this study**

2. **Figure S1**

3. **Results using alternative SDM assignment for species with mixed or equivocal SDM as listed in Table S1:**
   - **Table S2: MacroCAIC results using alternative SDM assignment**
   - **Table S3: BAMM estimation for the number of rate shifts in diversification. The number of rates shifts with the highest probability in each group is marked in bold.**
   - **Table S4: STRAPP results**
   - **Table S5: Summary of transition rate parameters estimates using the MK2 model with both Maximum Likelihood and Bayesian (MCMC) methodologies for the turtles, lizards, and squamate data sets using the alternative SDM assignment.**
   - **Table S6: Log likelihood differences (ΔLL) obtained between the single (BM1) and two rate (BM2) Brownian motion models of evolution, and between the single (OU1) and two (OU2) optimums, as estimated for lifepan in turtles, lizards and squamates. σ^2_{GSD} and σ^2_{TSD}, $Optimum_{GSD}$, and $Optimum_{TSD}$: estimated parameters for GSD and TSD lineages using the alternative SDM assignment.**

4. **BiSSE ANALYSES**

1. Table S1a: Dataset used in this study. Taxonomy follows Uetz and Hosek (2015)

ID	Vernacular	Order	Family	name.Uetz.March.2016	on.tree	SDM	SDMA	LIFESPAN	SourceGSD (karyotypic where available or incubation otherwise)	SourceTSD	NOTES (SDM data or taxon name on tree if different)
1	Tuatara	Rhyncocep	Sphenodontidae	Sphenodon punctatus	yes	TSD	TSD	91		Nelson et al. (2004)	
2	Turtle	Chelonia	Geoemydidae	Mauremys annamensis	yes	TSD	TSD	80		Ewert et al. (2004)	
3	Turtle	Chelonia	Geoemydidae	Mauremys japonica	yes	TSD	TSD	40		Okada Y. et al. (2010)	
4	Turtle	Chelonia	Geoemydidae	Mauremys mutica	yes	TSD	TSD	23.9		Ewert and Nelson (1991)	
5	Turtle	Chelonia	Geoemydidae	Mauremys nigricans	yes	TSD	TSD	16.2		Ewert et al. (2004)	
6	Turtle	Chelonia	Geoemydidae	Mauremys reevesii	yes	TSD	TSD	22.1		Ling H. (1985)	
7	Turtle	Chelonia	Geoemydidae	Melanochelys trijuga	yes	TSD	TSD	18		Ewert and Nelson (1991)	
8	Turtle	Chelonia	Geoemydidae	Pangshura smithii	yes	GSD	GSD	16.8	Sharma et al. (1975)		
9	Turtle	Chelonia	Geoemydidae	Rhinoclemmys areolata	yes	TSD	TSD	30		Ewert and Nelson (1991)	
10	Turtle	Chelonia	Geoemydidae	Rhinoclemmys pulcherrima	yes	TSD	TSD	20.4		Ewert and Nelson (1991)	
11	Turtle	Chelonia	Geoemydidae	Siebenrockiella crassicollis	yes	GSD	GSD	19.3	Carr and Bickham (1981)		
12	Turtle	Chelonia	Carettochelyidae	Carettochelys insculpta	yes	TSD	TSD	33		Webb et al. (1986)	
13	Turtle	Chelonia	Chelidae	Acanthochelys radiolata	yes	GSD	GSD	NA	McBee et al. (1985)		
14	Turtle	Chelonia	Chelidae	Chelodina longicollis	yes	GSD	GSD	38.4	Ezaz et al. (2006)		
15	Turtle	Chelonia	Chelidae	Elseya novaeguineae	yes	GSD	GSD	15.8	Ewert et al. (2004)		
16	Turtle	Chelonia	Chelidae	Elusor macrurus	yes	GSD	GSD	NA	Georges and McInnes. (1998)		
17	Turtle	Chelonia	Chelidae	Emydura macquarii	yes	GSD	GSD	20.9	Martinez et al. (2008)		
18	Turtle	Chelonia	Chelidae	Emydura subglobosa	yes	GSD	GSD	NA	Ewert and Nelson (1991)		
19	Turtle	Chelonia	Chelidae	Mesoclemmys gibba	yes	GSD	GSD	24.2	Ewert et al. (2004)		
20	Turtle	Chelonia	Chelidae	Phrynops geoffroanus	yes	GSD	GSD	20.1	Ewert et al. (2004)		
21	Turtle	Chelonia	Chelidae	Phrynops hilarii	yes	GSD	GSD	41	Ewert et al. (2004)		
22	Turtle	Chelonia	Cheloniidae	Caretta caretta	yes	TSD	TSD	25.2		Yntema and Mrosovsky (1979)	
23	Turtle	Chelonia	Cheloniidae	Chelonia mydas	yes	TSD	TSD	37		Mrosovsky et al. (1984)	
24	Turtle	Chelonia	Cheloniidae	Eretmochelys imbricata	yes	TSD	TSD	45		Mrosovsky et al. (1992)	
25	Turtle	Chelonia	Cheloniidae	Lepidochelys kempii	yes	TSD	TSD	40		Shaver D.J. et al. (1988)	
26	Turtle	Chelonia	Cheloniidae	Lepidochelys olivacea	yes	TSD	TSD	40		McCoy et al. (1983)	
27	Turtle	Chelonia	Cheloniidae	Natator depressus	yes	TSD	TSD	40		Hewavisenthi and Parmenter (2000)	
28	Turtle	Chelonia	Chelydridae	Chelydra serpentina	yes	TSD	TSD	50		Yntema C.L. (1976)	
29	Turtle	Chelonia	Chelydridae	Macrochelys temminckii	yes	TSD	TSD	18.2		Ewert and Nelson (1991)	
30	Turtle	Chelonia	Dermatemydidae	Dermatemys mawii	yes	TSD	TSD	11.3		Vogt and Flores-Villela (1992)	
31	Turtle	Chelonia	Dermochelyidae	Dermochelys coriacea	yes	TSD	TSD	NA		Rimblotet al. (1985)	
32	Turtle	Chelonia	Emydidae	Actinemys marmorata	yes	TSD	TSD	50		Ewertet al. (1994)	
33	Turtle	Chelonia	Emydidae	Chrysemys picta	yes	TSD	TSD	61		Ewert and Nelson (1991)	
34	Turtle	Chelonia	Emydidae	Clemmys guttata	yes	TSD	TSD	110		Ewert and Nelson (1991)	
35	Turtle	Chelonia	Emydidae	Deirochelys reticularia	yes	TSD	TSD	24		Ewert and Nelson (1991)	
36	Turtle	Chelonia	Emydidae	Emydoidea blandingii	yes	TSD	TSD	77		Ewert and Nelson (1991)	
37	Turtle	Chelonia	Emydidae	Emys orbicularis	yes	TSD	TSD	120		Pieau C. (1974)	
38	Turtle	Chelonia	Emydidae	Glyptemys insculpta	yes	GSD	GSD	100	Montiel et al. (2015)		
39	Turtle	Chelonia	Emydidae	Graptemys barbouri	yes	TSD	TSD	62.8		Ewert and Nelson (1991)	
40	Turtle	Chelonia	Emydidae	Graptemys geographica	yes	TSD	TSD	60		Ewert and Nelson (1991)	
41	Turtle	Chelonia	Emydidae	Graptemys nigrinoda	yes	TSD	TSD	31.7		Ewert and Nelson (1991)	
42	Turtle	Chelonia	Emydidae	Graptemys ouachitensis	yes	TSD	TSD	20		Ewert and Nelson (1991)	
43	Turtle	Chelonia	Emydidae	Graptemys pseudogeographica	yes	TSD	TSD	20.3		Ewert and Nelson (1991)	
44	Turtle	Chelonia	Emydidae	Graptemys pulchra	yes	TSD	TSD	15		Bull et al. (1982)	
45	Turtle	Chelonia	Emydidae	Graptemys versa	yes	TSD	TSD	35.4		Ewertet al. (1994)	
46	Turtle	Chelonia	Geoemydidae	Heosemys grandis	yes	TSD	TSD	20		Ewertet al. (1994)	
47	Turtle	Chelonia	Emydidae	Malaclemys terrapin	yes	TSD	TSD	40		Ewert and Nelson (1991)	
48	Turtle	Chelonia	Emydidae	Pseudemys concinna	yes	TSD	TSD	14.1		Ewert and Nelson (1991)	
49	Turtle	Chelonia	Emydidae	Pseudemys nelsoni	yes	TSD	TSD	23.4		Ewert et al. (2004)	
50	Turtle	Chelonia	Emydidae	Pseudemys peninsularis	yes	TSD	TSD	44		Ewert and Nelson (1991)	
51	Turtle	Chelonia	Emydidae	Pseudemys texana	yes	TSD	TSD	NA		Ewert et al. (2004)	
52	Turtle	Chelonia	Emydidae	Terrapene carolina	yes	TSD	TSD	23.9		Ewert and Nelson (1991)	
53	Turtle	Chelonia	Emydidae	Terrapene ornata	yes	TSD	TSD	54.8		Ewert and Nelson (1991)	
54	Turtle	Chelonia	Emydidae	Trachemys decorata	yes	TSD	TSD	127		Ewert et al. (2004)	

55	Turtle	Chelonia	Emydidae	Trachemys scripta	yes	TSD	TSD	13.9		Ewert and Nelson (1991)	
56	Turtle	Chelonia	Kinosternidae	Claudius angustatus	yes	GSD	GSD	16.1	Vogt and Flores-Villela (1992)		
57	Turtle	Chelonia	Kinosternidae	Kinosternon acutum	yes	TSD	TSD	NA		Janzen and Paukstis (1991)	
58	Turtle	Chelonia	Kinosternidae	Kinosternon alamosae	yes	TSD	TSD	29.6		Ewert et al. (2004)	
59	Turtle	Chelonia	Kinosternidae	Kinosternon arizonense	yes	TSD	TSD	25		Ewert and Nelson (1991)	
60	Turtle	Chelonia	Kinosternidae	Kinosternon baurii	yes	TSD	TSD	59.6		Ewert et al. (2004)	
61	Turtle	Chelonia	Kinosternidae	Kinosternon creaseri	yes	TSD	TSD	25		Ewert et al. (2004)	
62	Turtle	Chelonia	Kinosternidae	Kinosternon flavescens	yes	TSD	TSD	59.6		Ewertet al. (1994)	
63	Turtle	Chelonia	Kinosternidae	Kinosternon hirtipes	yes	TSD	TSD	NA		Ewertet al. (1994)	
64	Turtle	Chelonia	Kinosternidae	Kinosternon leucostomum	yes	TSD	TSD	40		Ewert and Nelson (1991)	
65	Turtle	Chelonia	Kinosternidae	Kinosternon scorpioides	yes	TSD	TSD	44.7		Ewert and Nelson (1991)	
66	Turtle	Chelonia	Kinosternidae	Kinosternon sonoriense	yes	TSD	TSD	44.7		Ewert et al. (2004)	
67	Turtle	Chelonia	Kinosternidae	Kinosternon subrubrum	yes	TSD	TSD	40		Ewert et al. (2004)	
68	Turtle	Chelonia	Kinosternidae	Staurotypus salvinii	yes	GSD	GSD	20.4	Ewert and Nelson (1991)		
69	Turtle	Chelonia	Kinosternidae	Staurotypus triporcatus	yes	GSD	GSD	16.7	Ewert and Nelson (1991)		
70	Turtle	Chelonia	Kinosternidae	Sternotherus carinatus	yes	TSD	TSD	26.3		Ewert and Nelson (1991)	
71	Turtle	Chelonia	Kinosternidae	Sternotherus minor	yes	TSD	TSD	33.5		Ewert and Nelson (1991)	
72	Turtle	Chelonia	Kinosternidae	Sternotherus odoratus	yes	TSD	TSD	29.3		Ewert and Nelson (1991)	
73	Turtle	Chelonia	Pelomedusidae	Pelomedusa subrufa	yes	TSD	TSD	13.8		Ewert and Nelson (1991)	
74	Turtle	Chelonia	Pelomedusidae	Pelusios castaneus	yes	TSD	TSD	24		Ewert and Nelson (1991)	
75	Turtle	Chelonia	Podocnemididae	Podocnemis erythrocephala	yes	TSD	TSD	37.5		Vogt R.C. (2001)	
76	Turtle	Chelonia	Podocnemididae	Podocnemis expansa	yes	TSD	TSD	37.4		Valenzuela N. (2001)	
77	Turtle	Chelonia	Podocnemididae	Podocnemis lewyana	yes	TSD	TSD	18		Paez et al. (2009)	
78	Turtle	Chelonia	Podocnemididae	Podocnemis sextuberculata	yes	TSD	TSD	25.3		Vogt R.C. (2008)	
79	Turtle	Chelonia	Podocnemididae	Podocnemis unifilis	yes	TSD	TSD	23.4		de Souza and Vogt (1994)	
80	Turtle	Chelonia	Testudinidae	Aldabrachelys gigantea	yes	TSD	TSD	152		Janzen and Paukstis (1991)	
81	Turtle	Chelonia	Testudinidae	Chelonoidis niger	yes	TSD	TSD	33.5		Janzen and Paukstis (1991)	
82	Turtle	Chelonia	Testudinidae	Gopherus agassizii	yes	TSD	TSD	177		Spotila et al. (1994)	
83	Turtle	Chelonia	Testudinidae	Gopherus polyphemus	yes	TSD	TSD	60		Demuth J.P. (2001)	
84	Turtle	Chelonia	Testudinidae	Testudo graeca	yes	TSD	TSD	138		Pieau C. (1971)	
85	Turtle	Chelonia	Testudinidae	Testudo hermanni	yes	TSD	TSD	50		Eendebak B.T. (1995)	
86	Turtle	Chelonia	Trionychidae	Apalone mutica	yes	GSD	GSD	20	Ewert and Nelson (1991)		
87	Turtle	Chelonia	Trionychidae	Apalone spinifera	yes	GSD	GSD	25.2	Badenhorst et al. (2013)		
88	Turtle	Chelonia	Trionychidae	Pelodiscus sinensis	yes	GSD	GSD	24	Kawai et al. (2007)		
89	lizard	Squamata	Agamidae	Agama agama	yes	TSD	TSD	8		Charnier M. (1966)	
90	lizard	Squamata	Agamidae	Agama impalearis	yes	TSD	TSD	6		El Mouden et al. (2001)	
91	lizard	Squamata	Agamidae	Amphibolurus muricatus	yes	TSD	TSD	4		Harlow (2004)	
92	lizard	Squamata	Agamidae	Amphibolurus norrisi	yes	GSD	GSD	7	Harlow (2004)		
93	lizard	Squamata	Agamidae	Calotes versicolor	yes	TSD	TSD	5		Inamdar et al. (2012)	
94	lizard	Squamata	Agamidae	Chlamydosaurus kingii	yes	TSD	TSD	15		Harlow (2004)	
95	lizard	Squamata	Agamidae	Ctenophorus decresii	yes	TSD	TSD	9		Harlow (2004)	
96	lizard	Squamata	Agamidae	Ctenophorus fordi	yes	GSD	GSD	2	Harlow (2004)		
97	lizard	Squamata	Agamidae	Ctenophorus ornatus	yes	TSD	GSD	11		Harlow (2004)	Weak TSD data (single temperature).
98	lizard	Squamata	Agamidae	Ctenophorus pictus	yes	TSD	GSD	NA	Uller et al. (2006)	Harlow (2004)	Strong data GSD. Weak TSD data.
99	lizard	Squamata	Agamidae	Diporiphora albilabris	yes	GSD	GSD	NA	Harlow (2004)		
100	lizard	Squamata	Agamidae	Diporiphora bilineata	yes	GSD	GSD	NA	Harlow (2004)		
101	lizard	Squamata	Agamidae	Diporiphora nobbi	yes	GSD	GSD	3.2	Harlow (2004)		
102	lizard	Squamata	Agamidae	Hypsilurus spinipes	yes	GSD	GSD	10	Harlow (2004)		
103	lizard	Squamata	Agamidae	Intellagama lesueurii	yes	TSD	TSD	28		Harlow (2004)	
104	lizard	Squamata	Agamidae	Lophognathus burnsi	no	TSD	GSD	NA		Harlow (2004)	Weak TSD data (Valenzuela 2004)
105	lizard	Squamata	Agamidae	Lophognathus gilberti	yes	TSD	TSD	NA		Harlow (2004)	
106	lizard	Squamata	Agamidae	Gowidon temporalis	yes	TSD	TSD	NA		Harlow (2004)	Lophognathus temporalis
107	lizard	Squamata	Agamidae	Paralaudakia caucasia	yes	TSD	TSD	13		Harlow (2004)	
108	lizard	Squamata	Agamidae	Phrynocephalus vlangalii	yes	GSD	GSD	NA	Zeng et al. (1997)		
109	lizard	Squamata	Agamidae	Pogona barbata	yes	GSD	GSD	13	Harlow (2004)		
110	lizard	Squamata	Agamidae	Pogona minor	yes	GSD	GSD	6	Harlow (2004)		
111	lizard	Squamata	Agamidae	Pogona vitticeps	yes	GSD	TSD	12	Ezaz et al. (2005)	Holleley et al. (2015	GSD+TSD
112	lizard	Squamata	Agamidae	Rankinia diemensis	yes	GSD	GSD	10	Harlow (2004)		

113	lizard	Squamata	Agamidae	Stellagama stellio	yes	TSD	TSD	10.4		Harlow (2004)	
114	lizard	Squamata	Agamidae	Tympanocryptis tetraporophora	yes	GSD	GSD	NA	Harlow (2004)		
115	lizard	Squamata	Anguidae	Elgaria multicarinata	yes	TSD	GSD	15	Telemeco RS. (2015)	Harlow (2004)	Weak data for each SDM.
116	lizard	Squamata	Carphodactylidae	Underwoodisaurus milii	yes	GSD	GSD	18	Pokorna et al. (2014)		
117	lizard	Squamata	Chamaeleonidae	Chamaeleo calyptratus	no	GSD	GSD	5	Andrews (2005)		
118	lizard	Squamata	Chamaeleonidae	Chamaeleo chamaeleon	yes	TSD	GSD	6	Andrews (2005)	Harlow (2004)	Small sample size.
119	lizard	Squamata	Chamaeleonidae	Furcifer lateralis	yes	TSD	GSD	3	Andrews (2005)	Harlow (2004)	
120	lizard	Squamata	Chamaeleonidae	Furcifer pardalis	yes	GSD	GSD	6	Viets et al. (1994)		
121	lizard	Squamata	Corytophanidae	Basiliscus plumifrons	yes	GSD	TSD	13	Viets et al. (1994)		Small sample size. No evidence for TSD. Weak GSD data.
122	lizard	Squamata	Crotaphytidae	Crotaphytus insularis	yes	GSD	GSD	NA	Rovatsos et al. (2014)		
123	lizard	Squamata	Dibamidae	Dibamus novaeguineae	yes	GSD	GSD	NA	Cole and Gans (1997)		
124	lizard	Squamata	Diplodactylidae	Correlophus ciliatus	yes	GSD	TSD	20	Gamble et al. (2015)	Harlow (2004)	GSD+TSD
125	lizard	Squamata	Diplodactylidae	Correlophus sarasinorum	yes	TSD	TSD	3.6		Harlow (2004)	
126	lizard	Squamata	Diplodactylidae	Mniarogekko chahoua	yes	TSD	TSD	5		Harlow (2004)	
127	lizard	Squamata	Diplodactylidae	Rhacodactylus auriculatus	yes	TSD	TSD	20		Harlow (2004)	
128	lizard	Squamata	Diplodactylidae	Rhacodactylus leachianus	yes	TSD	TSD	30		Harlow (2004)	
129	lizard	Squamata	Eublepharidae	Coleonyx brevis	yes	GSD	GSD	5	Pokorná et al. (2010)		
130	lizard	Squamata	Eublepharidae	Coleonyx elegans	yes	GSD	GSD	11	Pokorná et al. (2010)		
131	lizard	Squamata	Eublepharidae	Coleonyx mitratus	yes	GSD	GSD	NA	Kratochvil et al. (2008)		
132	lizard	Squamata	Eublepharidae	Coleonyx variegatus	yes	GSD	GSD	15.2	Pokorná et al. (2010)		
133	lizard	Squamata	Eublepharidae	Eublepharis macularius	yes	TSD	TSD	29		Viets et al. (1994)	
134	lizard	Squamata	Eublepharidae	Goniurosaurus kuroiwae	yes	TSD	TSD	9.4		Gamble (2010)	
135	lizard	Squamata	Eublepharidae	Goniurosaurus orientalis	no	TSD	TSD	NA		Gamble (2010)	
136	lizard	Squamata	Eublepharidae	Goniurosaurus splendens	no	TSD	TSD	NA		Gamble (2010)	
137	lizard	Squamata	Eublepharidae	Hemitheconyx caudicinctus	yes	TSD	TSD	16.2		Viets et al. (1994)	
138	lizard	Squamata	Gekkonidae	Christinus marmoratus	yes	GSD	GSD	12.8	King, M., Rofe R. (1976)		
140	lizard	Squamata	Gekkonidae	Dixonius siamensis	yes	GSD	GSD	NA	Ota et al. (2001)		
141	lizard	Squamata	Gekkonidae	Gehyra australis	yes	GSD	GSD	10	King M. (1983)		
142	lizard	Squamata	Gekkonidae	Gehyra mutilata	yes	GSD	GSD	9.25	Gamble et al. (2015)		
143	lizard	Squamata	Gekkonidae	Gehyra nana	yes	GSD	GSD	NA	Moritz, C. (1986)		
144	lizard	Squamata	Gekkonidae	Gehyra purpurascens	yes	GSD	GSD	NA	Moritz, C. (1984)		
145	lizard	Squamata	Gekkonidae	Gekko gecko	yes	GSD	GSD	23.5	Moritz C. (1990)		
146	lizard	Squamata	Gekkonidae	Gekko hokouensis	yes	GSD	GSD	NA	Shibaike Y. et al. (2009)		
147	lizard	Squamata	Gekkonidae	Gekko japonicus	yes	GSD	TSD	NA	Yoshida and Msahiro (1974)	Tokunaga, S. (1985)	GSD+TSD
148	lizard	Squamata	Gekkonidae	Hemidactylus frenatus	yes	GSD	GSD	7	Gamble et al. (2015)		
149	lizard	Squamata	Gekkonidae	Hemidactylus mabouia	yes	GSD	GSD	2.6	Gamble et al. (2015)		
150	lizard	Squamata	Gekkonidae	Hemidactylus platyurus	yes	GSD	GSD	NA	Trifonov et al. (2011)		
151	lizard	Squamata	Gekkonidae	Hemidactylus turcicus	yes	GSD	GSD	9	Gamble et al. (2015)		
152	lizard	Squamata	Gekkonidae	Hemidactylus vietnamensis	no	GSD	GSD	NA	Moritz C. (1990)		
153	lizard	Squamata	Gekkonidae	Heteronotia binoei	yes	GSD	GSD	13.6	Moritz C. (1990)		
154	lizard	Squamata	Gekkonidae	Lepidodactylus lugubris	yes	GSD	GSD	2.75	Volobouev and Pasteur (1988)		
155	lizard	Squamata	Gekkonidae	Paroedura karstophila	yes	GSD	GSD	NA	Koubová et al. (2014)		
156	lizard	Squamata	Gekkonidae	Paroedura lohatsara	yes	GSD	GSD	NA	Koubová et al. (2014)		
157	lizard	Squamata	Gekkonidae	Paroedura masobe	yes	GSD	GSD	NA	Koubová et al. (2014)		
158	lizard	Squamata	Gekkonidae	Paroedura oviceps	yes	GSD	GSD	NA	Koubová et al. (2014)		
159	lizard	Squamata	Gekkonidae	Paroedura picta	yes	GSD	GSD	5	Koubová et al. (2014)		
160	lizard	Squamata	Gekkonidae	Paroedura stumpffi	yes	GSD	GSD	NA	Koubová et al. (2014)		
161	lizard	Squamata	Gekkonidae	Phelsuma abbotti	yes	TSD	TSD	NA		Viets et al. (1994)	
162	lizard	Squamata	Gekkonidae	Phelsuma cepediana	yes	GSD	TSD	9.3	Viets et al. (1994)		Weak TSD data (Valenzuela 2004)
163	lizard	Squamata	Gekkonidae	Phelsuma dubia	yes	TSD	GSD	4.6		Viets et al. (1994)	Weak TSD data (Valenzuela 2004)
164	lizard	Squamata	Gekkonidae	Phelsuma grandis	no	TSD	TSD	20		Viets et al. (1994)	
165	lizard	Squamata	Gekkonidae	Phelsuma guentheri	yes	TSD	GSD	17.8		Viets et al. (1994)	Weak TSD data (Valenzuela 2004)
166	lizard	Squamata	Gekkonidae	Phelsuma guimbeaui	yes	TSD	GSD	8		Viets et al. (1994)	Weak TSD data (Valenzuela 2004)
167	lizard	Squamata	Gekkonidae	Phelsuma laticauda	yes	TSD	TSD	8.7		Viets et al. (1994)	
168	lizard	Squamata	Gekkonidae	Phelsuma lineata	yes	TSD	GSD	10		Viets et al. (1994)	Weak TSD data (Valenzuela 2004)
169	lizard	Squamata	Gekkonidae	Phelsuma madagascariensis	yes	TSD	GSD	13.6		Viets et al. (1994)	Weak TSD data (Valenzuela 2004).
170	lizard	Squamata	Gekkonidae	Phelsuma ornata	yes	GSD	TSD	NA	Viets et al. (1994)		Weak TSD data (Valenzuela 2004)

171	lizard	Squamata	Gekkonidae	Phelsuma pusilla	yes	TSD	TSD	NA		Viets et al. (1994)	
172	lizard	Squamata	Gekkonidae	Phelsuma sundbergi	yes	GSD	TSD	15.9	Viets et al. (1994)		Weak TSD data (Valenzuela 2004)
173	lizard	Squamata	Phyllodactylidae	Tarentola angustimentalis	yes	TSD	GSD	NA		Viets et al. (1994)	Weak TSD data (Valenzuela 2004)
174	lizard	Squamata	Phyllodactylidae	Tarentola annularis	yes	TSD	GSD	19.8		Viets et al. (1994)	Weak TSD data (Valenzuela 2004)
175	lizard	Squamata	Phyllodactylidae	Tarentola boettgeri	yes	TSD	TSD	6.1		Viets et al. (1994)	
176	lizard	Squamata	Phyllodactylidae	Tarentola delalandii	yes	TSD	GSD	7		Viets et al. (1994)	Weak TSD data (Valenzuela 2004)
177	lizard	Squamata	Phyllodactylidae	Tarentola gomerensis	yes	TSD	GSD	NA		Viets et al. (1994)	Weak TSD data (Valenzuela 2004)
178	lizard	Squamata	Phyllodactylidae	Tarentola mauritanica	yes	TSD	TSD	14		Viets et al. (1994)	
179	lizard	Squamata	Gymnophthalmida	Calyptommatus leiolepis	yes	GSD	GSD	NA	Yonenaga-Yassuda et al. (1998)		
180	lizard	Squamata	Gymnophthalmida	Calyptommatus nicterus	yes	GSD	GSD	NA	Yonenaga-Yassuda et al. (1998)		
181	lizard	Squamata	Gymnophthalmida	Calyptommatus sinebrachiatus	yes	GSD	GSD	NA	Yonenaga-Yassuda et al. (1998)		
182	lizard	Squamata	Gymnophthalmida	Gymnophthalmus pleei	yes	GSD	GSD	NA	Cole et al. (1990)		
183	lizard	Squamata	Gymnophthalmida	Micrablepharus atticolus	yes	GSD	GSD	NA	Yonenaga-Yassuda and Rodrigues (1999)		
184	lizard	Squamata	Gymnophthalmida	Micrablepharus maximiliani	yes	GSD	GSD	NA	Yonenaga-Yassuda and Rodrigues (1999)		
185	lizard	Squamata	Gymnophthalmida	Nothobachia ablephara	yes	GSD	GSD	NA	Pellegrino et al. (1999)		
186	lizard	Squamata	Helodermatidae	Heloderma suspectum	yes	GSD	GSD	43	Pokorná et al. (2014)		
187	lizard	Squamata	Dactyloidae	Anolis acutus	yes	GSD	GSD	NA	Gorman and Atkins (1969)		
188	lizard	Squamata	Dactyloidae	Anolis allisoni	yes	GSD	GSD	1.67	Rovatsos et al. (2014)		
189	lizard	Squamata	Dactyloidae	Anolis argenteolus	yes	GSD	GSD	NA	Rovatsos et al. (2014)		
190	lizard	Squamata	Dactyloidae	Anolis baracoae	yes	GSD	GSD	NA	Rovatsos et al. (2014)		
191	lizard	Squamata	Dactyloidae	Anolis barbatus	yes	GSD	GSD	NA	Rovatsos et al. (2014)		
192	lizard	Squamata	Dactyloidae	Anolis bartschi	yes	GSD	GSD	NA	Rovatsos et al. (2014)		
193	lizard	Squamata	Dactyloidae	Anolis bimaculatus	yes	GSD	GSD	7	Gorman (1965)		
194	lizard	Squamata	Dactyloidae	Anolis biporcatus	yes	GSD	GSD	3.1	Gorman (1973)		
195	lizard	Squamata	Dactyloidae	Anolis boulengerianus	yes	GSD	GSD	NA	Gamble et at. (2014)		
196	lizard	Squamata	Dactyloidae	Anolis brevirostris	yes	GSD	GSD	NA	Gamble et at. (2014)		
197	lizard	Squamata	Dactyloidae	Anolis carolinensis	yes	GSD	GSD	11	Viets et al. (1994)		
198	lizard	Squamata	Dactyloidae	Anolis caudalis	yes	GSD	GSD	NA	Gamble et at. (2014)		
199	lizard	Squamata	Dactyloidae	Anolis coelestinus	yes	GSD	GSD	NA	Rovatsos et al. (2014)		
200	lizard	Squamata	Dactyloidae	Anolis conspersus	yes	GSD	GSD	NA	Gorman and Atkins (1968)		
201	lizard	Squamata	Dactyloidae	Anolis cooki	yes	GSD	GSD	NA	Gorman et al. (1968)		
202	lizard	Squamata	Dactyloidae	Anolis crassulus	yes	GSD	GSD	NA	Gamble et at. (2014)		
203	lizard	Squamata	Dactyloidae	Anolis cristatellus	yes	GSD	GSD	6.9	Gorman et al. (1968)		
204	lizard	Squamata	Dactyloidae	Anolis desechensis	yes	GSD	GSD	NA	Gamble et at. (2014)		
205	lizard	Squamata	Dactyloidae	Anolis distichus	yes	GSD	GSD	NA	Gorman and Atkins (1969)		
206	lizard	Squamata	Dactyloidae	Anolis equestris	yes	GSD	GSD	16.5	Rovatsos et al. (2014)		
207	lizard	Squamata	Dactyloidae	Anolis evermanni	yes	GSD	GSD	NA	Gorman (1973)		
208	lizard	Squamata	Dactyloidae	Anolis ferreus	yes	GSD	GSD	NA	Gorman and Atkins (1969)		
209	lizard	Squamata	Dactyloidae	Anolis fuscoauratus	yes	GSD	GSD	NA	Rovatsos et al. (2014)		
210	lizard	Squamata	Dactyloidae	Anolis garmani	yes	GSD	GSD	10	Rovatsos et al. (2014)		
211	lizard	Squamata	Dactyloidae	Anolis gingivinus	yes	GSD	GSD	NA	Gorman and Atkins (1969)		
212	lizard	Squamata	Dactyloidae	Anolis grahami	yes	GSD	GSD	NA	Gorman (1973)		
213	lizard	Squamata	Dactyloidae	Anolis gundlachi	yes	GSD	GSD	3	Gorman (1973)		
214	lizard	Squamata	Dactyloidae	Anolis krugi	yes	GSD	GSD	NA	Gorman (1973)		
215	lizard	Squamata	Dactyloidae	Anolis leachii	yes	GSD	GSD	7	Gorman and Atkins (1969)		
216	lizard	Squamata	Dactyloidae	Anolis lineatopus	yes	GSD	GSD	1	Gamble et at. (2014)		
217	lizard	Squamata	Dactyloidae	Anolis lividus	yes	GSD	GSD	NA	Gamble et at. (2014)		
218	lizard	Squamata	Dactyloidae	Anolis luteogularis	yes	GSD	GSD	2.9	Rovatsos et al. (2014)		
219	lizard	Squamata	Dactyloidae	Anolis marmoratus	yes	GSD	GSD	NA	Gorman and Atkins (1969)		
220	lizard	Squamata	Dactyloidae	Anolis monensis	yes	GSD	GSD	NA	Gorman and Stamm (1975)		
221	lizard	Squamata	Dactyloidae	Anolis nebuloides	no	GSD	GSD	NA	Gamble et at. (2014)		
222	lizard	Squamata	Dactyloidae	Anolis nebulosus	no	GSD	GSD	NA	Gorman (1973)		
223	lizard	Squamata	Dactyloidae	Anolis nubilus	yes	GSD	GSD	NA	Gamble et at. (2014)		
224	lizard	Squamata	Dactyloidae	Anolis oculatus	yes	GSD	GSD	NA	Gorman and Atkins (1967)		
225	lizard	Squamata	Dactyloidae	Anolis onca	yes	GSD	GSD	NA	Olmo and Signorino (2005)		
226	lizard	Squamata	Dactyloidae	Anolis opalinus	yes	GSD	GSD	NA	Gorman (1973)		
227	lizard	Squamata	Dactyloidae	Anolis poncensis	yes	GSD	GSD	NA	Gorman (1973)		
228	lizard	Squamata	Dactyloidae	Anolis pulchellus	yes	GSD	GSD	NA	Gorman et al. (1968)		

229	lizard	Squamata	Dactyloidae	Anolis quercorum	yes	GSD	GSD	NA	Gamble et at. (2014)		
230	lizard	Squamata	Dactyloidae	Anolis roquet	yes	GSD	GSD	6.7	Rovatsos et al. (2014)		
231	lizard	Squamata	Dactyloidae	Anolis sabanus	yes	GSD	GSD	NA	Gorman and Atkins (1969)		
232	lizard	Squamata	Dactyloidae	Anolis sagrei	yes	GSD	GSD	8	de Smet (1978)		
233	lizard	Squamata	Dactyloidae	Anolis scriptus	yes	GSD	GSD	NA	Gorman et al. (1968)		
234	lizard	Squamata	Dactyloidae	Anolis stratulus	yes	GSD	GSD	1.6	Gorman and Atkins (1969)		
235	lizard	Squamata	Dactyloidae	Anolis trachyderma	yes	GSD	GSD	NA	Rovatsos et al. (2014)		
236	lizard	Squamata	Dactyloidae	Anolis wattsi	yes	GSD	GSD	NA	Gorman and Atkins (1969)		
237	lizard	Squamata	Dactyloidae	Anolis websteri	yes	GSD	GSD	NA	Gamble et at. (2014)		
238	lizard	Squamata	Corytophanidae	Basiliscus basiliscus	yes	GSD	GSD	9	Bohme W. (1975)		
239	lizard	Squamata	Crotaphytidae	Crotaphytus collaris	yes	GSD	GSD	10	de Smet (1978)		Weak TSD data (Valenzuela 2004)
240	lizard	Squamata	Iguanidae	Cyclura nubila	yes	GSD	GSD	54	Rovatsos et al. (2014)		
241	lizard	Squamata	Iguanidae	Dipsosaurus dorsalis	yes	GSD	TSD	14.6	Harlow (2004)		Weak TSD data (Valenzuela 2004)
242	lizard	Squamata	Tropiduridae	Eurolophosaurus amathites	yes	GSD	GSD	NA	Kasahara et al. (1987)		
243	lizard	Squamata	Tropiduridae	Eurolophosaurus nanuzae	yes	GSD	GSD	NA	Kasahara et al. (1987)		
244	lizard	Squamata	Iguanidae	Iguana iguana	yes	GSD	GSD	28	Rovatsos et al. (2014)		
245	lizard	Squamata	Liolaemidae	Phymaturus palluma	yes	GSD	GSD	12	Lamborot and Navarro-Suarez (1984)		
246	lizard	Squamata	Polychrotidae	Polychrus acutirostris	yes	GSD	GSD	NA	Peccinini et al. (1971)		
247	lizard	Squamata	Polychrotidae	Polychrus marmoratus	yes	GSD	GSD	NA	Gorman et al. (1967)		
248	lizard	Squamata	Polychrotidae	Polychrus peruvianus	no	GSD	GSD	NA	Gorman et al. (1969)		
249	lizard	Squamata	Leiosauridae	Pristidactylus achalensis	no	GSD	GSD	11	Gorman et al. (1967)		
250	lizard	Squamata	Phrynosomatidae	Sceloporus aeneus	yes	GSD	GSD	NA	Hall (1972)		
251	lizard	Squamata	Phrynosomatidae	Sceloporus anahuacus	no	GSD	GSD	NA	Leache and Sites (2009)		
252	lizard	Squamata	Phrynosomatidae	Sceloporus asper	no	GSD	GSD	NA	Hall (1972)		
253	lizard	Squamata	Phrynosomatidae	Sceloporus bulleri	yes	GSD	GSD	NA	Hall (1972)		
254	lizard	Squamata	Phrynosomatidae	Sceloporus cautus	yes	GSD	GSD	NA	Hall (1972)		
255	lizard	Squamata	Phrynosomatidae	Sceloporus chrysostictus	yes	GSD	GSD	NA	Gorman (1973)		
256	lizard	Squamata	Phrynosomatidae	Sceloporus clarkii	yes	GSD	GSD	NA	Cole (1970)		
257	lizard	Squamata	Phrynosomatidae	Sceloporus couchii	yes	GSD	GSD	NA	Hall (1972)		
258	lizard	Squamata	Phrynosomatidae	Sceloporus cozumelae	yes	GSD	GSD	NA	Cole (1978)		
259	lizard	Squamata	Phrynosomatidae	Sceloporus dugesii	yes	GSD	GSD	NA	Hall (1972)		
260	lizard	Squamata	Phrynosomatidae	Sceloporus gadoviae	yes	GSD	GSD	NA	Gorman (1973)		
261	lizard	Squamata	Phrynosomatidae	Sceloporus goldmani	yes	GSD	GSD	NA	Hall (1972)		
262	lizard	Squamata	Phrynosomatidae	Sceloporus graciosus	yes	GSD	GSD	8	Reed et al. (1990)		
263	lizard	Squamata	Phrynosomatidae	Sceloporus grammicus	yes	GSD	GSD	3	Hall and Selander (1973)		
264	lizard	Squamata	Phrynosomatidae	Sceloporus heterolepis	yes	GSD	GSD	NA	Hall (1972)		
265	lizard	Squamata	Phrynosomatidae	Sceloporus hunsakeri	yes	GSD	GSD	NA	Hall and Smith (1979)		
266	lizard	Squamata	Phrynosomatidae	Sceloporus jalapae	yes	GSD	GSD	NA	Hall (1972)		
267	lizard	Squamata	Phrynosomatidae	Sceloporus jarrovii	yes	GSD	GSD	2.8	Lowe et al. (1966)		
268	lizard	Squamata	Phrynosomatidae	Sceloporus licki	yes	GSD	GSD	NA	Hall and Smith (1979)		
269	lizard	Squamata	Phrynosomatidae	Sceloporus lundelli	yes	GSD	GSD	NA	Cole (1970)		
270	lizard	Squamata	Phrynosomatidae	Sceloporus maculosus	yes	GSD	GSD	NA	Cole (1971)		
271	lizard	Squamata	Phrynosomatidae	Sceloporus megalepidurus	yes	GSD	GSD	NA	Hall (1972)		
272	lizard	Squamata	Phrynosomatidae	Sceloporus melanorhinus	yes	GSD	GSD	NA	Cole (1970)		
273	lizard	Squamata	Phrynosomatidae	Sceloporus merriami	yes	GSD	GSD	6	Cole (1971)		
274	lizard	Squamata	Phrynosomatidae	Sceloporus mucronatus	yes	GSD	GSD	NA	Hall (1972)		
275	lizard	Squamata	Phrynosomatidae	Sceloporus nelsoni	yes	GSD	GSD	NA	Cole (1971)		
276	lizard	Squamata	Phrynosomatidae	Sceloporus occidentalis	yes	GSD	TSD	5	Viets et al. (1994)		Weak TSD data (Valenzuela 2004)
277	lizard	Squamata	Phrynosomatidae	Sceloporus orcutti	yes	GSD	GSD	16.25	Hall and Smith (1979)		
278	lizard	Squamata	Phrynosomatidae	Sceloporus ornatus	yes	GSD	GSD	NA	Hall (1972)		
279	lizard	Squamata	Phrynosomatidae	Sceloporus palaciosi	yes	GSD	GSD	NA	Leache and Sites (2009)		
280	lizard	Squamata	Phrynosomatidae	Sceloporus poinsettii	yes	GSD	GSD	8	Cole et al. (1967)		
281	lizard	Squamata	Phrynosomatidae	Sceloporus pyrocephalus	yes	GSD	GSD	NA	Cole (1971)		
282	lizard	Squamata	Phrynosomatidae	Sceloporus scalaris	yes	GSD	GSD	5	Cole (1978)		
283	lizard	Squamata	Phrynosomatidae	Sceloporus serrifer	yes	GSD	GSD	6.7	Hall (1973)		
284	lizard	Squamata	Phrynosomatidae	Sceloporus siniferus	yes	GSD	GSD	NA	Hall (1972)		
285	lizard	Squamata	Phrynosomatidae	Sceloporus spinosus	yes	GSD	GSD	NA	Hall (1972)		
286	lizard	Squamata	Phrynosomatidae	Sceloporus torquatus	yes	GSD	GSD	NA	Hall (1972)		

287	lizard	Squamata	Phrynosomatidae	Sceloporus undulatus	yes	GSD	GSD	4	Reed et al. (1990)		
288	lizard	Squamata	Phrynosomatidae	Sceloporus uniformis	no	GSD	GSD	NA	Gorman (1973)		
289	lizard	Squamata	Phrynosomatidae	Sceloporus variabilis	yes	GSD	GSD	1.5	Hall (1972)		
290	lizard	Squamata	Tropiduridae	Tropidurus hispidus	yes	GSD	GSD	2	Kasahara et al. (1987)		
291	lizard	Squamata	Tropiduridae	Tropidurus psammonastes	yes	GSD	GSD	NA	Rodrigues et al. (1988)		
292	lizard	Squamata	Tropiduridae	Tropidurus torquatus	yes	GSD	GSD	3	de Smet (1978)		
293	lizard	Squamata	Phrynosomatidae	Uma inornata	yes	GSD	GSD	5	Kral B. (1969)		
294	lizard	Squamata	Phrynosomatidae	Uta nolascensis	no	GSD	GSD	NA	Pennock et al. (1969)		
295	lizard	Squamata	Phrynosomatidae	Uta palmeri	yes	GSD	GSD	NA	Pennock et al. (1969)		
296	lizard	Squamata	Phrynosomatidae	Uta squamata	yes	GSD	GSD	NA	Pennock et al. (1969)		
297	lizard	Squamata	Phrynosomatidae	Uta stansburiana	yes	GSD	GSD	4.8	Pennock et al. (1969)		
298	lizard	Squamata	Lacertidae	Acanthodactylus erythrurus	yes	GSD	GSD	NA	Olmo et al. (1987)		
299	lizard	Squamata	Lacertidae	Algyroides moreoticus	yes	GSD	GSD	9.2	Odierna et al. (1993)		
300	lizard	Squamata	Lacertidae	Algyroides nigropunctatus	yes	GSD	GSD	2.25	Odierna et al. (1993)		
301	lizard	Squamata	Lacertidae	Darevskia armeniaca	yes	GSD	GSD	8	Olmo et al. (1990)		
302	lizard	Squamata	Lacertidae	Darevskia dahli	no	GSD	GSD	6	Kupriyanova (1992)		
303	lizard	Squamata	Lacertidae	Darevskia mixta	yes	GSD	GSD	NA	Kupriyanova (1992)		
304	lizard	Squamata	Lacertidae	Darevskia raddei	yes	GSD	GSD	6	Kupriyanova (1989)		
305	lizard	Squamata	Lacertidae	Darevskia rostombekowi	yes	GSD	GSD	6	Kupriyanova (1989)		
306	lizard	Squamata	Lacertidae	Darevskia unisexualis	no	GSD	GSD	7	Kupriyanova (1989)		
307	lizard	Squamata	Lacertidae	Darevskia valentini	yes	GSD	GSD	7	Kupriyanova (1989)		
308	lizard	Squamata	Lacertidae	Eremias arguta	yes	GSD	GSD	NA	Olmo et al. (1990)		
309	lizard	Squamata	Lacertidae	Eremias grammica	yes	GSD	GSD	NA	Kupriyanova (1994)		
310	lizard	Squamata	Lacertidae	Eremias multiocellata	yes	GSD	TSD	NA	Tang et al. (2012)	Zhang et al. (2010)	GSD+TSD
311	lizard	Squamata	Lacertidae	Eremias velox	yes	GSD	GSD	NA	Olmo et al. (1990)		
312	lizard	Squamata	Lacertidae	Gallotia galloti	yes	GSD	GSD	15	Olmo (1986)		
313	lizard	Squamata	Lacertidae	Heliobolus lugubris	yes	GSD	GSD	NA	Odierna et al. (1990)		
314	lizard	Squamata	Lacertidae	Hellenolacerta graeca	yes	GSD	GSD	NA	Olmo et al. (1987)		
315	lizard	Squamata	Lacertidae	Iberolacerta aranica	yes	GSD	GSD	NA	Odierna et al. (2001)		
316	lizard	Squamata	Lacertidae	Iberolacerta aurelioi	yes	GSD	GSD	16	Odierna et al. (1996)		
317	lizard	Squamata	Lacertidae	Iberolacerta cyreni	yes	GSD	GSD	NA	Odierna et al. (1996)		
318	lizard	Squamata	Lacertidae	Iberolacerta horvathi	yes	GSD	GSD	NA	Capula M. et al. (1989)		
319	lizard	Squamata	Lacertidae	Iberolacerta monticola	yes	GSD	GSD	15	Rojo et al. (2012)		
320	lizard	Squamata	Lacertidae	Lacerta agilis	yes	GSD	GSD	12	de Smet (1981)		
321	lizard	Squamata	Lacertidae	Lacerta bilineata	yes	GSD	GSD	13	Olmo et al. (1985)		
322	lizard	Squamata	Lacertidae	Lacerta strigata	yes	GSD	GSD	4	Ivanov and Fedorova (1970)		
323	lizard	Squamata	Lacertidae	Lacerta trilineata	yes	GSD	GSD	7.75	Gorman (1969)		
324	lizard	Squamata	Lacertidae	Lacerta viridis	yes	GSD	GSD	10	de Smet (1981)		
325	lizard	Squamata	Lacertidae	Meroles cuneirostris	yes	GSD	GSD	NA	Olmo et al. (1987)		
326	lizard	Squamata	Lacertidae	Mesalina olivieri	yes	GSD	GSD	5	Gorman (1969)		
327	lizard	Squamata	Lacertidae	Omanosaura jayakari	yes	GSD	GSD	17	Fritz B. et al. (1991)		
328	lizard	Squamata	Lacertidae	Ophisops elegans	yes	GSD	GSD	6	Bhatnagar and Yoniss (1976)		
329	lizard	Squamata	Lacertidae	Pedioplanis namaquensis	yes	GSD	GSD	NA	Odierna et al. (1990)		
330	lizard	Squamata	Lacertidae	Phoenicolacerta kulzeri	yes	GSD	GSD	NA	Bosch et al. (2003)		
331	lizard	Squamata	Lacertidae	Phoenicolacerta laevis	yes	GSD	GSD	4.55	Bosch et al. (2003)		
332	lizard	Squamata	Lacertidae	Podarcis erhardii	yes	GSD	GSD	5	Olmo et al. (1990)		
333	lizard	Squamata	Lacertidae	Podarcis hispanicus	yes	GSD	GSD	13	Odierna et al. (1990)		
334	lizard	Squamata	Lacertidae	Podarcis melisellensis	yes	GSD	GSD	3.9	de Smet (1981)		
335	lizard	Squamata	Lacertidae	Podarcis muralis	yes	GSD	GSD	10.1	Viets et al. (1994)		
336	lizard	Squamata	Lacertidae	Podarcis pityusensis	yes	TSD	GSD	18		Harlow (2004)	Weak TSD data (Valenzuela 2004)
337	lizard	Squamata	Lacertidae	Podarcis siculus	yes	GSD	GSD	3.7	Olmo et al. (1990)		
338	lizard	Squamata	Lacertidae	Podarcis tiliguerta	yes	GSD	GSD	15	Olmo et al. (1990)		
339	lizard	Squamata	Lacertidae	Podarcis waglerianus	no	GSD	GSD	NA	Capriglione et al. (1994)		
340	lizard	Squamata	Lacertidae	Psammodromus algirus	yes	GSD	GSD	2.75	de Smet (1981)		
341	lizard	Squamata	Phrynosomatidae	Sceloporus parvus	yes	GSD	GSD		Cole (1978)		
342	lizard	Squamata	Lacertidae	Takydromus sexlineatus	yes	GSD	GSD	1.5	Olmo et al. (1984)		
343	lizard	Squamata	Lacertidae	Teira dugesii	yes	GSD	GSD	16	Olmo and Signorino (2005)		
344	lizard	Squamata	Lacertidae	Timon lepidus	yes	GSD	GSD	34	Olmo et al. (1987)		

345	lizard	Squamata	Lacertidae	Zootoca vivipara	yes	GSD	GSD	12	Chevalier et al. (1979)		
346	lizard	Squamata	Leiocephalidae	Leiocephalus carinatus	yes	GSD	GSD	10.8	Rovatsos et al. (2014)		
347	lizard	Squamata	Opluridae	Chalarodon madagascariensis	yes	GSD	GSD	NA	Rovatsos et al. (2014)		
348	lizard	Squamata	Opluridae	Oplurus fierinensis	yes	GSD	GSD	NA	Rovatsos et al. (2014)		
349	lizard	Squamata	Phrynosomatidae	Petrosaurus thalassinus	yes	GSD	GSD	20	Rovatsos et al. (2014)		
350	lizard	Squamata	Phrynosomatidae	Sceloporus malachiticus	yes	GSD	GSD	NA	Rovatsos et al. (2014)		
351	lizard	Squamata	Diplodactylidae	Oedura marmorata	yes	GSD	GSD	21.2	Moritz C. (1990)		
352	lizard	Squamata	Phyllodactylidae	Phyllodactylus lanei	yes	GSD	GSD	NA	Moritz C. (1990)		
353	lizard	Squamata	Phyllodactylidae	Thecadactylus rapicauda	yes	GSD	GSD	6.9	Schmid et al. (2014)		
354	lizard	Squamata	Pygopodidae	Aprasia parapulchella	yes	GSD	GSD	NA	Matsubara et al. (2013)		
355	lizard	Squamata	Pygopodidae	Delma inornata	yes	GSD	GSD	5	King M. (1990)		
356	lizard	Squamata	Pygopodidae	Lialis burtonis	yes	GSD	GSD	NA	Gorman and Gress (1970)		
357	lizard	Squamata	Scincidae	Bassiana duperreyi	yes	GSD	TSD	7	Quinn et al. (2009)	Shine et al. (2002)	GSD+TSD
358	lizard	Squamata	Scincidae	Eulamprus heatwolei	yes	TSD	TSD	NA		Langkilde and Shine (2005)	
359	lizard	Squamata	Scincidae	Eulamprus tympanum	yes	TSD	TSD	15		Valenzuela et al. (2003)	
360	lizard	Squamata	Scincidae	Mabuya mabouya	yes	GSD	GSD	NA	Becak et al. (1972)		
361	lizard	Squamata	Scincidae	Niveoscincus ocellatus	yes	TSD	TSD	12		Wapstra et al. (2004)	
362	lizard	Squamata	Scincidae	Oligosoma maccanni	yes	GSD	GSD	NA	Hare et al. (2011)		
363	lizard	Squamata	Scincidae	Oligosoma oliveri	yes	GSD	GSD	NA	Hardy G.S. (1979)		
364	lizard	Squamata	Scincidae	Oligosoma suteri	yes	GSD	GSD	12	Hare et al. (2012)		
365	lizard	Squamata	Scincidae	Plestiodon fasciatus	yes	GSD	GSD	4.9	Viets et al. (1994)		
366	lizard	Squamata	Scincidae	Plestiodon obsoletus	yes	GSD	GSD	7.33	Viets et al. (1994)		
367	lizard	Squamata	Scincidae	Pseudemoia baudini	no	GSD	GSD	NA	Hutchinson and Donnellan (1992)		
368	lizard	Squamata	Scincidae	Pseudemoia cryodroma	no	GSD	GSD	NA	Hutchinson and Donnellan (1992)		
369	lizard	Squamata	Scincidae	Pseudemoia entrecasteauxii	yes	GSD	GSD	5	Hutchinson and Donnellan (1992)		
370	lizard	Squamata	Scincidae	Pseudemoia pagenstecheri	yes	GSD	GSD	NA	Hutchinson and Donnellan (1992)		
371	lizard	Squamata	Scincidae	Pseudemoia rawlinsoni	no	GSD	GSD	NA	Hutchinson and Donnellan (1992)		
372	lizard	Squamata	Scincidae	Pseudemoia spenceri	no	GSD	GSD	NA	Hutchinson and Donnellan (1992)		
373	lizard	Squamata	Scincidae	Saproscincus czechurai	yes	GSD	GSD	NA	Donnellan (1991)		
374	lizard	Squamata	Scincidae	Scincella lateralis	yes	GSD	GSD	4	Olmo (1986)		
375	lizard	Squamata	Scincidae	Sphenomorphus indicus	yes	TSD	TSD	6		Ji et al. (2006)	
376	lizard	Squamata	Sphaerodactylidae	Aristelliger expectatus	no	GSD	GSD	NA	Gamble et al. (2015)		
377	lizard	Squamata	Sphaerodactylidae	Gonatodes ceciliae	yes	GSD	GSD	NA	McBee et al. (1987)		
378	lizard	Squamata	Sphaerodactylidae	Sphaerodactylus macrolepis	yes	GSD	GSD	NA	Gamble et al. (2015)		
379	lizard	Squamata	Sphaerodactylidae	Sphaerodactylus nicholsi	yes	GSD	GSD	NA	Gamble et al. (2015)		
380	lizard	Squamata	Teiidae	Aspidoscelis inornata	yes	GSD	GSD	NA	Viets et al. (1994)		
381	lizard	Squamata	Teiidae	Aspidoscelis tigris	yes	GSD	GSD	8	Cole et al. (1969)		
382	lizard	Squamata	Teiidae	Aspidoscelis uniparens	no	GSD	GSD	4	Viets et al. (1994)		
383	lizard	Squamata	Tropiduridae	Uranoscodon superciliosus	yes	GSD	GSD	4.8	Rovatsos et al. (2014)		
384	lizard	Squamata	Varanidae	Varanus acanthurus	yes	GSD	GSD	10	Olmo (1986)		
385	lizard	Squamata	Varanidae	Varanus albigularis	yes	GSD	GSD	16.7	King and King (1975)		
386	lizard	Squamata	Varanidae	Varanus exanthematicus	yes	GSD	GSD	17	King and King (1975)		
387	lizard	Squamata	Varanidae	Varanus gouldii	yes	GSD	GSD	18.3	Matsubara et al. (2014)		
388	lizard	Squamata	Varanidae	Varanus komodoensis	yes	GSD	GSD	62	Sulandari et al. (2014)		
389	lizard	Squamata	Varanidae	Varanus niloticus	yes	GSD	GSD	14.6	King and King (1975)		
390	lizard	Squamata	Varanidae	Varanus rosenbergi	yes	GSD	GSD	NA	Matsubara et al. (2014)		
391	lizard	Squamata	Varanidae	Varanus salvator	yes	TSD	GSD	15.7		Harlow (2004)	Weak TSD data (Valenzuela 2004)
392	lizard	Squamata	Varanidae	Varanus varius	yes	GSD	GSD	22	King and King (1975)		
393	snake	Squamata	Boidae	Acrantophis dumerili	yes	GSD	GSD	26	Mengden and Stock (1980)		
394	snake	Squamata	Boidae	Boa constrictor	yes	GSD	GSD	40.4	Olmo (2005)		
395	snake	Squamata	Pythonidae	Liasis olivaceus	yes	GSD	GSD	NA	Mengden and Stock (1980)		
396	snake	Squamata	Pythonidae	Morelia spilota	yes	GSD	GSD	19.6			
397	snake	Squamata	Boidae	Sanzinia madagascariensis	yes	GSD	GSD	21.8	Mengden and Stock (1980)		
398	snake	Squamata	Pythonidae	Simalia boeleni	yes	GSD	GSD	20.1	Mengden and Stock (1980)		
399	snake	Squamata	Colubridae	Ahaetulla nasuta	yes	GSD	GSD	NA	Sharma and Nakhasi (1979)		
400	snake	Squamata	Natricidae	Amphiesma stolatum	yes	GSD	GSD	NA	Ray-Chaudhuri et al. (1971)		
401	snake	Squamata	Colubridae	Argyrogena fasciolata	no	GSD	GSD	NA	Ray-Chaudhuri et al. (1971)		
402	snake	Squamata	Colubridae	Bogertophis subocularis	yes	GSD	GSD	23.8	Baker et al. (1971)		

403	snake	Squamata	Colubridae	Boiga forsteni	yes	GSD	GSD	NA	Ray-Chaudhuri et al. (1971)		
404	snake	Squamata	Colubridae	Boiga trigonata	yes	GSD	GSD	8.2	Ray-Chaudhuri et al. (1971)		
405	snake	Squamata	Homalopsidae	Cerberus rynchops	yes	GSD	GSD	NA	Singh, L. (1972)		
406	snake	Squamata	Colubridae	Chironius bicarinatus	yes	GSD	GSD	NA	Becak (1965)		
407	snake	Squamata	Colubridae	Chironius quadricarinatus	yes	GSD	GSD	NA	Becak et al. (1966)		
408	snake	Squamata	Colubridae	Chrysopelea ornata	yes	GSD	GSD	4.3	Sharma and Nakhasi (1979)		
409	snake	Squamata	Dipsadidae	Clelia clelia	yes	GSD	GSD	11.5	Becak (1965)		
410	snake	Squamata	Colubridae	Coelognathus radiatus	yes	GSD	GSD	NA	Singh et al. (1979)		
411	snake	Squamata	Colubridae	Dendrelaphis punctulatus	no	GSD	GSD	18	Mengden (1982)		
412	snake	Squamata	Colubridae	Drymarchon corais	yes	GSD	GSD	25.9	Becak (1965)		
413	snake	Squamata	Colubridae	Drymarchon couperi	no	GSD	GSD	25.45	Becak et al. (1964)		
414	snake	Squamata	Colubridae	Elaphe climacophora	yes	GSD	GSD	13.1	Itoh et al. (1970)		
415	snake	Squamata	Colubridae	Elaphe quadrivirgata	yes	GSD	GSD	16.8	Itoh et al. (1970)		
416	snake	Squamata	Dipsadidae	Erythrolamprus almadensis	yes	GSD	GSD	NA	Becak et al. (1975)		
417	snake	Squamata	Dipsadidae	Erythrolamprus miliaris	yes	GSD	GSD	NA	Becak et al. (1966)		
418	snake	Squamata	Colubridae	Euprepiophis conspicillata	yes	GSD	GSD	NA	Toriba (1990)		
419	snake	Squamata	Dipsadidae	Geophis omiltemanus	no	GSD	GSD	NA	Hardy (1976)		
420	snake	Squamata	Homalopsidae	Gerarda prevostiana	yes	GSD	GSD	NA	Singh et al. (1970)		
421	snake	Squamata	Natricidae	Hebius pryeri	no	GSD	GSD	NA	Toriba (1990)		
422	snake	Squamata	Natricidae	Hebius vibakari	no	GSD	GSD	NA	Toriba (1990)		
423	snake	Squamata	Dipsadidae	Hydromorphus concolor	yes	GSD	GSD	NA	Solorzano et al. (1989)		
424	snake	Squamata	Colubridae	Lycodon aulicus	yes	GSD	GSD	NA	Nakamura K. (1935)		
425	snake	Squamata	Colubridae	Lycodon semicarinatus	yes[AN]	GSD	GSD	NA	Toriba (1990)		Dinodon semicarinatum
426	snake	Squamata	Natricidae	Macropisthodon rudis	yes	GSD	GSD	NA	Nakamura K. (1935)		
427	snake	Squamata	Colubridae	Mastigodryas bifossatus	yes	GSD	GSD	NA	Becak (1965)		
428	snake	Squamata	Natricidae	Natrix maura	yes	GSD	GSD	9.2	Kobel (1967)		
429	snake	Squamata	Natricidae	Natrix natrix	yes	GSD	GSD	20	Kobel (1967)		
430	snake	Squamata	Natricidae	Natrix tessellata	yes	GSD	GSD	14	de Smet (1978)		
431	snake	Squamata	Dipsadidae	Oxyrhopus petolarius	yes	GSD	GSD	NA	Becak (1969)		
432	snake	Squamata	Colubridae	Pantherophis alleghaniensis	yes	GSD	GSD	NA	Baker et al. (1971)		
433	snake	Squamata	Colubridae	Pantherophis obsoletus	yes	GSD	GSD	33.9	Mengden and Stock (1980)		
434	snake	Squamata	Dipsadidae	Philodryas aestiva	yes	GSD	GSD	NA	Becak (1969)		
435	snake	Squamata	Dipsadidae	Philodryas chamissonis	no	GSD	GSD	NA	Moreno et al. (1987)		
436	snake	Squamata	Dipsadidae	Philodryas olfersii	yes	GSD	GSD	NA	Becak (1965)		
437	snake	Squamata	Dipsadidae	Philodryas patagoniensis	yes	GSD	GSD	NA	Becak (1969)		
438	snake	Squamata	Dipsadidae	Pseudoboa nigra	yes	GSD	GSD	NA	Becak et al. (1975)		
439	snake	Squamata	Colubridae	Ptyas mucosa	yes	GSD	GSD	11.3	Ray-Chaudhuri et al. (1971)		
440	snake	Squamata	Natricidae	Rhabdophis tigrinus	yes	GSD	GSD	NA	Itoh et al. (1970)		
441	snake	Squamata	Dipsadidae	Rhachidelus brazili	yes	GSD	GSD	NA	Becak et al. (1975)		
442	snake	Squamata	Natricidae	Sinonatrix annularis	yes	GSD	GSD	NA	Rossman and Eberle (1977)		
443	snake	Squamata	Natricidae	Sinonatrix percarinata	yes	GSD	GSD	NA	Rossman and Eberle (1977)		
444	snake	Squamata	Colubridae	Spilotes pullatus	yes	GSD	GSD	17.5	Becak (1965)		
445	snake	Squamata	Natricidae	Storeria dekayi	yes	GSD	GSD	7	Baker et al. (1972)		
446	snake	Squamata	Dipsadidae	Thamnodynastes hypoconia	yes	GSD	GSD	NA	Becak (1969)		
447	snake	Squamata	Dipsadidae	Thamnodynastes strigatus	yes	GSD	GSD	NA	Becak (1969)		
448	snake	Squamata	Natricidae	Thamnophis marcianus	yes	GSD	GSD	7	Mengden and Stock (1980)		
449	snake	Squamata	Dipsadidae	Tomodon dorsatum	yes	GSD	GSD	NA	Becak et al. (1966)		
450	snake	Squamata	Dipsadidae	Tropidodryas serra	yes	GSD	GSD	NA	Becak (1969)		
451	snake	Squamata	Natricidae	Tropidonophis mairii	no	GSD	GSD	NA	Mengden (1981)		
452	snake	Squamata	Natricidae	Xenochrophis piscator	yes	GSD	GSD	9	Singh et al. (1968)		
453	snake	Squamata	Dipsadidae	Xenodon merremi	yes[AN]	GSD	GSD	NA	Becak (1965)		Waglerophis merremii
454	snake	Squamata	Dipsadidae	Xenodon neuwiedii	yes	GSD	GSD	NA	Becak (1969)		
455	snake	Squamata	Colubridae	Zamenis longissimus	yes	GSD	GSD	3.55	de Smet (1978)		
456	snake	Squamata	Elapidae	Acanthophis antarcticus	yes	GSD	GSD	9.3	Mengden (1982)		
457	snake	Squamata	Elapidae	Acanthophis praelongus	yes	GSD	GSD	NA	Mengden (1982)		
458	snake	Squamata	Elapidae	Acanthophis pyrrhus	no	GSD	GSD	3.4	Mengden (1982)		
459	snake	Squamata	Elapidae	Aipysurus fuscus	yes	GSD	GSD	NA	Mengden (1982)		
460	snake	Squamata	Elapidae	Aipysurus laevis	yes	GSD	GSD	NA	Mengden (1982)		

461	snake	Squamata	Elapidae	Antaioserpens warro	no	GSD	GSD	NA	Mengden (1982)		
462	snake	Squamata	Elapidae	Austrelaps superbus	yes	GSD	GSD	13	Mengden (1982)		
463	snake	Squamata	Elapidae	Bungarus caeruleus	yes	GSD	GSD	17.4	Singh et al. (1970)		
464	snake	Squamata	Elapidae	Bungarus fasciatus	yes	GSD	GSD	13.2	Singh et al. (1974)		
465	snake	Squamata	Elapidae	Bungarus sindanus	yes	GSD	GSD	NA	Singh et al. (1980)		
466	snake	Squamata	Elapidae	Cacophis squamulosus	yes	GSD	GSD	NA	Mengden (1982)		
467	snake	Squamata	Elapidae	Cryptophis boschmai	no	GSD	GSD	NA	Mengden (1982)		
468	snake	Squamata	Elapidae	Cryptophis nigrescens	yes[AN]	GSD	GSD	NA	Mengden (1982)		Rhinoplocephalus nigrescens
469	snake	Squamata	Elapidae	Cryptophis nigrostriatus	no	GSD	GSD	NA	Mengden (1982)		
470	snake	Squamata	Elapidae	Demansia olivacea	no	GSD	GSD	NA	Mengden (1982)		
471	snake	Squamata	Elapidae	Demansia papuensis	yes	GSD	GSD	NA	Mengden (1982)		
472	snake	Squamata	Elapidae	Demansia psammophis	yes	GSD	GSD	NA	Mengden (1982)		
473	snake	Squamata	Elapidae	Demansia torquata	no	GSD	GSD	NA	Mengden (1982)		
474	snake	Squamata	Elapidae	Demansia vestigiata	yes	GSD	GSD	NA	Mengden (1982)		
475	snake	Squamata	Elapidae	Dendroaspis polylepis	yes	GSD	GSD	26.2	Mengden (1982)		
476	snake	Squamata	Elapidae	Denisonia devisi	yes	GSD	GSD	26	Mengden (1982)		
477	snake	Squamata	Elapidae	Drysdalia coronoides	yes	GSD	GSD	NA	Mengden (1982)		
478	snake	Squamata	Elapidae	Drysdalia mastersii	yes	GSD	GSD	NA	Mengden (1982)		
479	snake	Squamata	Elapidae	Drysdalia rhodogaster	no	GSD	GSD	NA	Mengden (1982)		
480	snake	Squamata	Elapidae	Echiopsis curta	yes	GSD	GSD	NA	Mengden (1982)		
481	snake	Squamata	Elapidae	Elapognathus minor	no	GSD	GSD	NA	Mengden (1982)		
482	snake	Squamata	Elapidae	Emydocephalus annulatus	yes	GSD	GSD	NA	Mengden (1982)		
483	snake	Squamata	Elapidae	Furina diadema	yes	GSD	GSD	NA	Mengden (1982)		
484	snake	Squamata	Elapidae	Hemiaspis signata	yes	GSD	GSD	NA	Mengden (1982)		
485	snake	Squamata	Elapidae	Hoplocephalus bitorquatus	yes	GSD	GSD	NA	Mengden (1982)		
486	snake	Squamata	Elapidae	Hydrophis cantoris	no	GSD	GSD	NA	Singh et al. (1972)		
487	snake	Squamata	Elapidae	Hydrophis fasciatus	no	GSD	GSD	NA	Ray-Chaudhuri et al. (1972)		
488	snake	Squamata	Elapidae	Hydrophis gracilis	no	GSD	GSD	NA	Singh et al. (1972)		
489	snake	Squamata	Elapidae	Hydrophis jerdonii	no	GSD	GSD	NA	Singh et al. (1974)		
490	snake	Squamata	Elapidae	Hydrophis major	yes[AN]	GSD	GSD	NA	Mengden (1982)		Disteira major
491	snake	Squamata	Elapidae	Hydrophis ornatus	yes	GSD	GSD	NA	Singh et al. (1972)		
492	snake	Squamata	Elapidae	Hydrophis platurus	yes[AN]	GSD	GSD	NA	Gutierrez and Bolanos. (1980)		Pelamis platura
493	snake	Squamata	Elapidae	Hydrophis schistosus	yes	GSD	GSD	NA	Ray-Chaudhuri et al. (1971)		
494	snake	Squamata	Elapidae	Hydrophis spiralis	yes	GSD	GSD	NA	Ray-Chaudhuri et al. (1971)		
495	snake	Squamata	Elapidae	Laticauda colubrina	yes	GSD	GSD	NA	Gorman (1981)		
496	snake	Squamata	Elapidae	Laticauda laticaudata	yes	GSD	GSD	NA	Gorman (1981)		
497	snake	Squamata	Elapidae	Laticauda semifasciata	no	GSD	GSD	NA	Gorman (1981)		
498	snake	Squamata	Elapidae	Micrurus browni	no	GSD	GSD	NA	Gutierrez et al. (1988)		
499	snake	Squamata	Elapidae	Micrurus diastema	yes	GSD	GSD	NA	Gutierrez et al. (1988)		
500	snake	Squamata	Elapidae	Micrurus elegans	no	GSD	GSD	NA	Gutierrez et al. (1988)		
501	snake	Squamata	Elapidae	Micrurus fulvius	yes	GSD	GSD	19	Graham (1977)		
502	snake	Squamata	Elapidae	Micrurus hippocrepis	no	GSD	GSD	NA	Gutierrez et al. (1988)		
503	snake	Squamata	Elapidae	Micrurus lemniscatus	yes	GSD	GSD	NA	Becak and Becak (1969)		
504	snake	Squamata	Elapidae	Micrurus mosquitensis	no	GSD	GSD	NA	Gutierrez and Bolanos. (1979)		
505	snake	Squamata	Elapidae	Micrurus nigrocinctus	no	GSD	GSD	NA	Gutierrez and Bolanos. (1979)		
506	snake	Squamata	Elapidae	Micrurus surinamensis	yes	GSD	GSD	NA	Gutierrez et al. (1988)		
507	snake	Squamata	Elapidae	Naja kaouthia	yes	GSD	GSD	25.4	Singh et al. (1970)		
508	snake	Squamata	Elapidae	Naja melanoleuca	yes	GSD	GSD	30	Mengden (1982)		
509	snake	Squamata	Elapidae	Naja naja	yes	GSD	GSD	32.3	Singh et al. (1970)		
510	snake	Squamata	Elapidae	Naja nigricollis	yes	GSD	GSD	23.2	de Smet (1978)		
511	snake	Squamata	Elapidae	Naja oxiana	no	GSD	GSD	NA	Singh et al. (1980)		
512	snake	Squamata	Elapidae	Notechis scutatus	yes	GSD	GSD	17	Shine and Bull (1977)		
513	snake	Squamata	Elapidae	Oxyuranus scutellatus	yes	GSD	GSD	15.6	Mengden (1982)		
514	snake	Squamata	Elapidae	Parasuta dwyeri	no	GSD	GSD	NA	Mengden (1982)		
515	snake	Squamata	Elapidae	Parasuta flagellum	no	GSD	GSD	NA	Mengden (1982)		
516	snake	Squamata	Elapidae	Parasuta gouldii	no	GSD	GSD	NA	Mengden (1982)		
517	snake	Squamata	Elapidae	Parasuta monachus	yes[AN]	GSD	GSD	NA	Mengden (1982)		Suta monachus

518	snake	Squamata	Elapidae	Parasuta nigriceps	no	GSD	GSD	NA	Mengden (1982)		
519	snake	Squamata	Elapidae	Pseudechis australis	yes	GSD	GSD	11.1	Mengden (1982)		
520	snake	Squamata	Elapidae	Pseudechis butleri	yes	GSD	GSD	NA	Mengden (1982)		
521	snake	Squamata	Elapidae	Pseudechis guttatus	yes	GSD	GSD	NA	Mengden (1982)		
522	snake	Squamata	Elapidae	Pseudechis porphyriacus	yes	GSD	GSD	25	Mengden (1982)		
523	snake	Squamata	Elapidae	Pseudonaja affinis	no	GSD	GSD	NA	Mengden (1982)		
524	snake	Squamata	Elapidae	Pseudonaja nuchalis	no	GSD	GSD	NA	Mengden (1982)		
525	snake	Squamata	Elapidae	Pseudonaja textilis	yes	GSD	GSD	15	Mengden (1982)		
526	snake	Squamata	Elapidae	Simoselaps bertholdi	yes	GSD	GSD	NA	Mengden (1982)		
527	snake	Squamata	Elapidae	Suta punctata	no	GSD	GSD	NA	Mengden (1982)		
528	snake	Squamata	Elapidae	Suta suta	yes	GSD	GSD	12	Mengden (1982)		
529	snake	Squamata	Elapidae	Tropidechis carinatus	yes	GSD	GSD	9	Mengden (1982)		
530	snake	Squamata	Pythonidae	Simalia amethistina	yes[AN]	GSD	GSD	13.8	Mengden and Stock (1980)		Morelia amethistina
531	snake	Squamata	Viperidae	Agkistrodon contortrix	yes	GSD	GSD	29.8	Baker et al. (1972)		
532	snake	Squamata	Viperidae	Atropoides nummifer	yes	GSD	GSD	19	Gutierrez and Bolanos. (1979)		
533	snake	Squamata	Viperidae	Atropoides picadoi	yes	GSD	GSD	13	Gutierrez and Bolanos. (1979)		
534	snake	Squamata	Viperidae	Bothriechis lateralis	yes	GSD	GSD	12.5	Gutierrez and Bolanos. (1979)		
535	snake	Squamata	Viperidae	Bothriechis nigroviridis	yes	GSD	GSD	NA	Gutierrez and Bolanos. (1979)		
536	snake	Squamata	Viperidae	Bothriechis schlegelii	yes	GSD	GSD	19.5	de Smet (1978)		
537	snake	Squamata	Viperidae	Bothrops alternatus	yes	GSD	GSD	15.2	Becak (1965)		
538	snake	Squamata	Viperidae	Bothrops asper	yes	GSD	GSD	20.4	Gutierrez and Bolanos. (1979)		
539	snake	Squamata	Viperidae	Bothrops insularis	yes	GSD	GSD	NA	Becak et al. (1990)		
540	snake	Squamata	Viperidae	Bothrops jararaca	yes	GSD	GSD	6.5	Becak et al. (1962)		
541	snake	Squamata	Viperidae	Bothrops jararacussu	yes	GSD	GSD	NA	Becak et al. (1990)		
542	snake	Squamata	Viperidae	Bothrops leucurus	yes	GSD	GSD	NA	Becak (1965)		
543	snake	Squamata	Viperidae	Bothrops moojeni	yes	GSD	GSD	NA	Becak et al. (1964)		
544	snake	Squamata	Viperidae	Bothrops neuwiedi	yes	GSD	GSD	15.1	Becak et al. (1990)		
545	snake	Squamata	Viperidae	Cerrophidion godmani	yes	GSD	GSD	15.8	Gutierrez and Bolanos. (1979)		
546	snake	Squamata	Viperidae	Crotalus atrox	yes	GSD	GSD	27	Stewar et al.(1990)		
547	snake	Squamata	Viperidae	Crotalus catalinensis	yes	GSD	GSD	11.8	Stewar et al.(1990)		
548	snake	Squamata	Viperidae	Crotalus cerastes	yes	GSD	GSD	27.3	Ohno, S. (1967)		
549	snake	Squamata	Viperidae	Crotalus durissus	yes	GSD	GSD	19.8	Becak (1965)		
550	snake	Squamata	Viperidae	Crotalus enyo	yes	GSD	GSD	17.1	Stewar et al.(1990)		
551	snake	Squamata	Viperidae	Crotalus molossus	yes	GSD	GSD	20.7	Baker et al. (1971)		
552	snake	Squamata	Viperidae	Crotalus ruber	yes	GSD	GSD	19.2	Stewar et al.(1990)		
553	snake	Squamata	Viperidae	Crotalus scutulatus	yes	GSD	GSD	14.4	Stewar et al.(1990)		
554	snake	Squamata	Viperidae	Daboia russelii	yes	GSD	GSD	15	Ray-Chaudhuri and Singh (1972)		
555	snake	Squamata	Viperidae	Echis carinatus	yes	GSD	GSD	23.8	Singh et al. (1970)		
556	snake	Squamata	Viperidae	Lachesis muta	yes	GSD	GSD	31.6	Becak and Becak (1969)		
557	snake	Squamata	Viperidae	Macrovipera lebetina	yes	GSD	GSD	13.3	de Smet (1978)		
558	snake	Squamata	Viperidae	Porthidium nasutum	yes	GSD	GSD	NA	Gutierrez and Bolanos. (1979)		
559	snake	Squamata	Viperidae	Vipera ammodytes	yes	GSD	GSD	22	Saint Girons (1977)		
560	snake	Squamata	Viperidae	Vipera aspis	yes	GSD	GSD	25	Kobel (1967)		
561	snake	Squamata	Viperidae	Vipera berus	yes	GSD	GSD	19	Kobel (1967)		
562	snake	Squamata	Viperidae	Vipera latastei	yes	GSD	GSD	14	Saint Girons (1977)		
563	snake	Squamata	Viperidae	Vipera monticola	no	GSD	GSD	NA	Saint Girons (1977)		
564	snake	Squamata	Viperidae	Vipera renardi	yes	GSD	GSD	NA	Saint Girons (1977)		
565	snake	Squamata	Viperidae	Vipera seoanei	yes	GSD	GSD	NA	Saint Girons (1977)		
566	snake	Squamata	Viperidae	Vipera ursinii	yes	GSD	GSD	NA	Kobel (1967)		

REFERENCES
Andrews RM. (2005) Incubation temperature and sex ratio of the veiled chameleon (Chamaeleo calyptratus). J Herpetol 39: 515-518.
Badenhorst, D., R. Stanyon, T. Engstrom, and N. Valenzuela (2013) A ZZ/ZW microchromosome system in the spiny softshell turtle, Apalone spinifera, reveals an intriguing sex chromosome conservation in Trionychidae. Chrom. Res. 21:137-147.
Baker R.J. et al. (1971) ex Olmo E., Signorino G.G. (2005) ChromoRep: A reptiles chromosomes database.
Baker R.J. et al. (1972) ex Olmo E., Signorino G.G. (2005) ChromoRep: A reptiles chromosomes database.
Becak & Becak (1969) ex Olmo E., Signorino G.G. (2005) ChromoRep: A reptiles chromosomes database.
Becak M.L. (1965) ex Olmo E., Signorino G.G. (2005) ChromoRep: A reptiles chromosomes database.
Becak M.L. (1969) ex Olmo E., Signorino G.G. (2005) ChromoRep: A reptiles chromosomes database.
Becak M.L. et al. (1990) The W chromosome during the evolution and in sex abnormalities of snakes. DNA content, C-banding., Pages 221-240 in E. Olmo, ed. Cytogenetics of Amphibians and Reptiles. Berlin, Birkhauser Verlag Basel.
Becak W. et al. (1962) ex Olmo E., Signorino G.G. (2005) ChromoRep: A reptiles chromosomes database.
Becak W. et al. (1964) ex Olmo E., Signorino G.G. (2005) ChromoRep: A reptiles chromosomes database.
Becak W. et al. (1966) ex Olmo E., Signorino G.G. (2005) ChromoRep: A reptiles chromosomes database.
Becak W. et al. (1972) ex Olmo E., Signorino G.G. (2005) ChromoRep: A reptiles chromosomes database.
Becak W. et al. (1975) ex Olmo E., Signorino G.G. (2005) ChromoRep: A reptiles chromosomes database.
Bhatnagar & Yoniss (1976) ex Olmo E., Signorino G.G. (2005) ChromoRep: A reptiles chromosomes database.
Bohme W. (1975) Indizien fur naturliche Parthenogenese beim Helmbasilisken, Basiliscus basiliscus (Linnaeus 1758). Salamandra 11: 77-83.
Bosch I.D.H. et al. (2003) Karyological and genetic variation in Middle Eastern lacertid lizards, Lacerta laevis and the Lacerta kulzeri complex: a case of chromosomal allopatric speciation. Chromosome Research 11: 165-178.
Bull J.J., Vogt R.C., McCoy C.J. (1982) Sex determining temperatures in turtles: a geographic comparison. Evolution 36: 326-332.
Capriglione T., Olmo E., Odierna G., Kupriyanova L.A. (1994) Mechanisms of differentiation in the sex chromosomes of some Lacertidae Amphibia-Reptilia 15: 1-8.
Capula M. et al. (1989) ex Olmo E., Signorino G.G. (2005) ChromoRep: A reptiles chromosomes database.
Carr, J.L., Bickham, J.W. (1981) Sex-Chromosomes of the Asian Black Pond Turtle, Siebenrockiella crassicollis (Testudines, Emydidae). Cytogenetics and Cell Genetics 31: 178-183.
Charnier M. (1966) Action de la temperature sur la sex-ratio chez l'embryon d'Agama agama (Agamidae, Lacertilien
Chevalier M. et al. (1979) ex Pokorna M., Kratochvil L. (2009) Phylogeny of sex-determining mechanisms in squamate reptiles: are sex chromosomes an evolutionary trap? Zoological Journal of the Linnean Society 156: 168-183.
Cole & Gans (1997) ex Olmo E. (2005) Rate of chromosome changes and speciation in reptiles. Genetica 125, 185-203.
Cole C.J. (1970) Karyotypes and evolution of the spinosus group of lizards in the genus Sceloporus. Am. Mus. Novit. No. 2431: 1-47.
Cole C.J. (1971) ex Olmo E., Signorino G.G. (2005) ChromoRep: A reptiles chromosomes database.
Cole C.J. (1971) Karyotypes of the five monotypic species groups of lizards in the genus Sceloporus. American Mus Novit No. 2450: 1-17.
Cole C.J. (1978) ex Olmo E., Signorino G.G. (2005) ChromoRep: A reptiles chromosomes database.
Cole C.J. (1978) Karyotypes and systematics of the lizards in the Sceloporus variabilis, Sceloporus jalapae and Sceloporus scalaris species groups. American Museum Novitates: 1-13.
Cole C.J. et al. (1967) ex Olmo E., Signorino G.G. (2005) ChromoRep: A reptiles chromosomes database.
Cole C.J. et al. (1990) ex Olmo E., Signorino G.G. (2005) ChromoRep: A reptiles chromosomes database.
Cole C.J., C.H. Lowe, and J.W. Wright. (1969) Sex chromosomes in teiid whiptail lizards (genus Cnemidophorus). Am. Mus. Novit. No. 2395:1-14.
de Smet W.H.O. (1978) Chromosomes of 22 species of Chelonia (Reptilia). Acta Zoologica et Pathologica Antverpiensia, 15-34.
de Smet W.H.O. (1978) ex Olmo E., Signorino G.G. (2005) ChromoRep: A reptiles chromosomes database.
de Smet W.H.O. (1981) ex Olmo E., Signorino G.G. (2005) ChromoRep: A reptiles chromosomes database.
de Souza R.R., Vogt R.C. (1994) Incubation temperature influences sex and hatchling size in the neotropical turtle Podocnemis unifilis. J. Herpetol. 28: 453-464.
Demuth J.P. (2001) The effects of constant and fluctuating incubation temperatures on sex determination, growth, and performance in the tortoise Gopherus polyphemus. Can. J. Zool. 79: 1609-1620.
Donnellan S.C. (1991) ex Olmo E., Signorino G.G. (2005) ChromoRep: A reptiles chromosomes database.
Eendebak B.T. (1995) Incubation period and sex ratio of Hermann's tortoise, Testudo hermanni boettgeri. Chelonian Conservation and Biology 1: 227-231.
El Mouden E.H., Znari M., Pieau C. (2001) Effects of Incubation Temperature on Embryonic Development and Sex Determination in the North African Agamid Lizard, Agama impalearis. Herpetological Journal 11: 101-108.
Ewert M.A., Etchberger C.R., Nelson C.E. (2004) Turtle sex determining modes and TSD patterns, and some TSD pattern correlates. In: Temperature Dependent Sex Determination in Vertebrates (eds. Valenzuela N, Lance VA), pp. 21-32. Smithsonian Books, Washington, DC.
Ewert M.A., Jackson D.R., Nelson C.E. (1994) Patterns of temperature-dependent sex determination in turtles. The Journal of Experimental Zoology 270: 3-15.
Ewert M.A., Nelson C.E. (1991) Sex determination in turtles: diverse patterns and some possible adaptive values. Copeia 1991, 50-69.
Ezaz, T., N. Valenzuela, F. Grutzner, I. Miura, A. Georges, R. L. Burke, and J. A. M. Graves. (2006) An XX/XY sex microchromosome system in a freshwater turtle, Chelodina longicollis (Testudines : Chelidae) with genetic sex determination. Chromosome Research 14: 139-150.
Fritz B. et al. (1991) ex Olmo E., Signorino G.G. (2005) ChromoRep: A reptiles chromosomes database.
Gamble, T. 2010. A Review of Sex Determining Mechanisms in Geckos (Gekkota: Squamata). Sex. Dev. 4:88-103.
Gamble, T., A. J. Geneva, R. E. Glor, and D. Zarkower. (2014) Anolis sex chromosomes are derived from a single ancestral pair. Evolution 68:1027-1041.
Gamble, T., J. Coryell, T. Ezaz, J. Lynch, D. P. Scantlebury, and D. Zarkower. (2015) Restriction Site-Associated DNA Sequencing (RAD-seq) Reveals an Extraordinary Number of Transitions among Gecko Sex-Determining Systems. Mol. Biol. Evol. 32:1296-1309.
Georges, A., and S. McInnes. (1998) Temperature fails to influence hatchling sex in another genus and species of chelid turtle, Elusor macrurus. Journal of Herpetology 32: 596-598.
Gorman & Atkins (1968) ex Olmo E., Signorino G.G. (2005) ChromoRep: A reptiles chromosomes database.
Gorman & Atkins (1969) ex Olmo E., Signorino G.G. (2005) ChromoRep: A reptiles chromosomes database.
Gorman & Gress (1970) ex Olmo E., Signorino G.G. (2005) ChromoRep: A reptiles chromosomes database.
Gorman G.C. (1965) ex Olmo E., Signorino G.G. (2005) ChromoRep: A reptiles chromosomes database.
Gorman G.C. (1969) ex Olmo E., Signorino G.G. (2005) ChromoRep: A reptiles chromosomes database.
Gorman G.C. (1973) The chromosomes of the Reptilia, a cytotaxonomic interpretation. In: Cytotaxonomy and vertebrate evolution (eds. Chiarelli AB, Capanna E), pp. 349-424. Academic Press, New York.
Gorman G.C. et al. (1968) ex Olmo E., Signorino G.G. (2005) ChromoRep: A reptiles chromosomes database.
Gorman G.C. et al. (1969) ex Olmo E., Signorino G.G. (2005) ChromoRep: A reptiles chromosomes database.
Gorman G.C., Atkins L. (1967) Relationships of Anolis of Roquet species group (Sauria - Iguanidae) .2. Comparative chromosome cytology. Systematic Zoology 16: 137-143.
Gorman G.C., Atkins L. (1969) The zoo geography of lesser Antillean Anolis lizards an analysis based upon chromosomes and lactic dehydrogenases. Bulletin of the Museum of Comparative Zoology 138: 53-80.
Gorman G.C., Atkins L., Holzinger T. (1967) New karyotype data on 15 genera of lizards in the family Iguanidae, with a discussion of taxonomic and cytological implications. Cytogenetics 6: 286-299.
Gorman, G.C. (1981) The chromosomes of Laticauda and a review of karyotypic evolution in the Elapidae. Journal of Herpetology 15: 225-233.
Gorman, G.C., Stamm. B. (1975) The Anolis lizards of Mona, Redonda, and La Blanquilla: chromosomes, relationships, and natural history notes. Journal Herpet 9: 197-205.
Graham, G.L. (1977) The Karyotype of the Texas Coral Snake, Micrurus fulvius tenere. Herpetologica 33: 345-348.
Gutierrez J.M., Bolanos R. (1979) Cariotipos de las principales serpientes coral (Elapidae: Micrurus) de Costa Rica. Revista De Biologia Tropical 27: 57-73.
Gutierrez, J. M., and R. Bolanos. (1980) Karyotype of the yellow-bellied sea-snake, Pelamis platurus. Journal of Herpetology 14: 161-165.
Gutierrez, J.M., A. Solorzano, L. Cerdas, and J.P. Vannini. (1988) Karyotypes of Five Species of Coral Snakes (Micrurus). Journal of Herpetology 22: 109-112.
Hall & Smith (1979) ex Olmo E., Signorino G.G. (2005) ChromoRep: A reptiles chromosomes database.
Hall W.P. (1972) unpublished manuscript ex Olmo E., Signorino G.G. (2005) ChromoRep: A reptiles chromosomes database.
Hall W.P. (1973) ex Olmo E., Signorino G.G. (2005) ChromoRep: A reptiles chromosomes database.
Hall, W.P., Selander R.K. (1973) Hybridization of karyotypically differentiated populations in the Sceloporus grammicus complex (Iguanidae). Evolution 27: 226-242.

Hardy G.S. (1979) ex Olmo E., Signorino G.G. (2005) ChromoRep: A reptiles chromosomes database.

Hardy L.M. (1976) ex Olmo E., Signorino G.G. (2005) ChromoRep: A reptiles chromosomes database.

Hare, K.M., C. Yeong, and A. Cree. (2011) Does Gestational Temperature or Prenatal Sex Ratio Influence Development of Sexual Dimorphism in a Viviparous Skink? Journal of Experimental Zoology Part A-Ecological Genetics and Physiology 315A: 215-221.

Hare, K.M., C.H. Daugherty, and A. Cree. (2002) Incubation regime affects juvenile morphology and hatching success, but not sex, of the oviparous lizard Oligosoma suteri (Lacertilia : Scinidae). New Zealand Journal of Zoology 29: 221-229.

Harlow PS (2004) Temperature-Dependent Sex Determination in Lizards. In: Temperature Dependent Sex Determination in Vertebrates (eds. Valenzuela N, Lance VA), pp. 42-52. Smithsonian Books, Washington, DC.

Hewavisenthi S., Parmenter C.J. (2000) Hydric environment and sex determination in the flatback turtle (Natator depressus Garman

Holleley, C. E., O'Meally, D., Sarre, S. D., Marshall Graves, J. A., Ezaz, T., Matsubara, K., Azad, B., Zhang, X. & Georges, A. (2015) Sex reversal triggers the rapid transition from genetic to temperature-dependent sex. Nature 523: 79-82.

Hutchinson, M.N., Donnellan S.C. (1992) Taxonomy and genetic variation in the australian lizards of the genus Pseudemoia (Scincidae, Lygosominae). Journal of Natural History 26: 215-264.

Inamdar L.S., Vani V., Seshagiri P.B. (2012) A tropical oviparous lizard, Calotes versicolor, exhibiting a potentially novel FMFM pattern of temperature-dependent sex determination. Journal of Experimental Zoology Part A-Ecological Genetics and Physiology 317A: 32-46.

Itoh M. et al. (1970) ex Olmo E., Signorino G.G. (2005) ChromoRep: A reptiles chromosomes database.

Ivanov & Fedorova (1970) ex Olmo E., Signorino G.G. (2005) ChromoRep: A reptiles chromosomes database.

Janzen F.J., Paukstis G.L. (1991) Environmental sex determination in reptiles: ecology, evolution and experimental design. The Quaterly Review of Biology 66: 149-179.

Ji X. et al. (2006) Gestation temperature affects sexual phenotype, morphology, locomotor performance, and growth of neonatal brown forest skinks, Sphenomorphus indicus. Biological Journal of the Linnean Society 88: 453-463.

Kasahara S. et al. (1987) ex Olmo E., Signorino G.G. (2005) ChromoRep: A reptiles chromosomes database.

Kawai, A., C. Nishida-Umehara, J. Ishijima, Y. Tsuda, H. Ota, and Y. Matsuda. (2007) Different origins of bird and reptile sex chromosomes inferred from comparative mapping of chicken Z-linked genes. Cytogenetic and Genome Research 117: 92-102.

King and King (1975) ex Olmo E., Signorino G.G. (2005) ChromoRep: A reptiles chromosomes database.

King M. (1983) Karyotypic evolution in Gehyra (Gekkonidae, Reptilia) .3. The Gehyra australis complex. Australian Journal of Zoology 31: 723-741.

King M. (1990) in Olmo (ed) Cytogenetics of Amphibians and Reptiles. Birkhauser, Basel, Boston, Berlin. pp 153-180.

King M., King D. (1975) Chromosomal evolution in the lizard genus Varanus (Reptilia). Aus. J. Biol. Sci. 28: 89-108.

King, M., Rofe R. (1976) Karyotypic variation in australian gekko Phyllodactylus marmoratus (Gray) (Gekkonidae, Reptilia). Chromosoma 54: 75-87.

Kobel H.R. (1967) ex Olmo E., Signorino G.G. (2005) ChromoRep: A reptiles chromosomes database.

Koubová, M., M. Pokorná, M. Rovatsos, K. Farkačová, M. Altmanová, and L. Kratochvíl. 2014. Sex determination in Madagascar geckos of the genus Paroedura (Squamata: Gekkonidae): are differentiated sex chromosomes indeed so evolutionary stable? Chrom. Res. 22:441-452.

Kral B. (1969) ex Olmo E., Signorino G.G. (2005) ChromoRep: A reptiles chromosomes database.

Kratochvil, L., L. Kubicka, E. Landova. (2008) Does the mechanism of sex determination constrain the potential for sex manipulation? A test in geckos with contrasting sex-determining systems. Naturwissenschaften 95 :209-215.

Kupriyanova L.A. (1989) ex Olmo E., Signorino G.G. (2005) ChromoRep: A reptiles chromosomes database.

Kupriyanova L.A. (1992) ex Olmo E., Signorino G.G. (2005) ChromoRep: A reptiles chromosomes database.

Kupriyanova L.A. (1994) ex Olmo E., Signorino G.G. (2005) ChromoRep: A reptiles chromosomes database.

Lamborot & Navarro-Suarez (1984) ex Olmo E., Signorino G.G. (2005) ChromoRep: A reptiles chromosomes database.

Langkilde T., Shine R. (2005) Different optimal offspring sizes for sons and daughters may favor the evolution of temperature-dependent sex determination in viviparous lizards. Evolution 59: 2275-2280.

Leache, A.D., Sites, Jr, J.W. (2009) Chromosome Evolution and Diversification in North American Spiny Lizards (Genus Sceloporus). Cytogenetic and Genome Research 127: 166-181.

Ling H. (1985) Sex determination by temperature for incubation in Chinemys reeversii. Acta Herpetologica Sinica 4: 130.

Lowe C.H. et al. (1966) ex Olmo E., Signorino G.G. (2005) ChromoRep: A reptiles chromosomes database.

Martinez, P., N. Valenzuela, A. Georges, and J. A. M. Graves. (2008) An XX/XY heteromorphic sex chromosome system in the Australian chelid turtle Emydura macquarii, a new piece in the puzzle of sex chromosome evolution in turtles. Chromosome Research 16: 815-825.

Matsubara, K., S. D. Sarre, A. Georges, Y. Matsuda, J. A. M. Graves, and T. Ezaz. (2014) Highly Differentiated ZW Sex Microchromosomes in the Australian Varanus Species Evolved through Rapid Amplification of Repetitive Sequences. Plos One 9.

Matsubara, K., T. Knopp, S. D. Sarre, A. Georges, and T. Ezaz. (2013) Karyotypic analysis and FISH mapping of microsatellite motifs reveal highly differentiated XX/XY sex chromosomes in the pink-tailed worm-lizard (Aprasia parapulchella, Pygopodidae, Squamata). Molecular Cytogenetics 6.

McBee, K., Bickham J.W., Dixon, J.R. (1987) Male Heterogamety and Chromosomal Variation in Caribbean Geckos. Journal of Herpetology 21: 68-71.

McBee, K., J. W. Bickham, A. G. J. Rhodin, and R. A. Mittermeier. (1985) Karyotypic Variation in the Genus Platemys (Testudines, Pleurodira). Copeia 1985: 445-449.

McCoy C.J., Vogt R.C., Censky E.J. (1983) Temperature-controlled sex determination in the sea turtle Lepidochelys olivacea. J. Herpetol. 17: 404-406.

Mengden G.A. (1981) ex Olmo E., Signorino G.G. (2005) ChromoRep: A reptiles chromosomes database.

Mengden G.A. (1982) ex Olmo E., Signorino G.G. (2005) ChromoRep: A reptiles chromosomes database.

Mengden, G.A., Stock, A.S. (1980) Chromosomal evolution in serpentes; comparison of G and C chromosome banding patterns in some colubrid and boid genera. Chromosoma 79: 53-64.

Moreno R. et al. (1987) ex Olmo E., Signorino G.G. (2005) ChromoRep: A reptiles chromosomes database.

Moritz C. (1990) Patterns and process of sex chromosome evolution in gekkonid lizards (Sauria: Reptilia). In: Cytogenetics of Amphibians and Reptiles, pp. 205-219. Birkhauser Verlag Basel.

Moritz, C. 1984. The evolution of a highly variable sex-chromosome in Gehyra purpurascens (Gekkonidae). Chromosoma 90:111-119.

Moritz, C. 1986. The population biology of gehyra gekkonidae chromosome change and speciation. Systematic Zoology 35:46-67.

Mrosovsky N., Bass A., Corliss L.A., Richardson J.I., Richardson T.H. (1992) Pivotal and beach temperatures for hawksbill turtles nesting in Antigua. Can. J. Zool. 70: 1920-1925.

Mrosovsky N., Dutton P.H., Whitmore C.P. (1984) Sex ratios of two species of sea turtle nesting Suriname. Can. J. Zool 62: 2227-2239.

Nakamura K. (1935) ex Olmo E., Signorino G.G. (2005) ChromoRep: A reptiles chromosomes database.

Nelson N.J., Cree A., Thompson M.B., Keall S.N., Daugherty C.H. (2004) Temperature-dependent Sex Determination in Tuatara. In: Temperature dependent sex determination in vertebrates (eds. Valenzuela N, Lance VA)

Odierna G. et al. (1990) ex Olmo E., Signorino G.G. (2005) ChromoRep: A reptiles chromosomes database.

Odierna G. et al. (1993) ex Olmo E., Signorino G.G. (2005) ChromoRep: A reptiles chromosomes database.

Odierna G. et al. (1996) ex Olmo E., Signorino G.G. (2005) ChromoRep: A reptiles chromosomes database.

Odierna G. et al. (2001) ex Olmo E., Signorino G.G. (2005) ChromoRep: A reptiles chromosomes database.

Ohno, S. (1967) Sex chromosomes and sex-linked genes: Monographs on Endocrinology. Berlin, Springer-Verlag.

Okada Y. et al. (2010) Temperature-dependent Sex Determination in the Japanese Pond Turtle, Mauremys japonica (Reptilia: Geoemydidae

Olmo E. (1986) Reptilia, Pages 94 in J. Bernard, ed. Animal cytogenetics. Berlin, Germany, Gebruder Borntraeger.

Olmo E. (2005) Rate of chromosome changes and speciation in reptiles. Genetica 125: 185-203.

Olmo E. et al. (1985) ex Olmo E., Signorino G.G. (2005) ChromoRep: A reptiles chromosomes database.

Olmo E. et al. (1987) ex Olmo E., Signorino G.G. (2005) ChromoRep: A reptiles chromosomes database.

Olmo E., Signorino G.G. (2005) ChromoRep: A reptiles chromosomes database.

Olmo, E., G. Odierna, T. Capriglione, and A. Cardone. (1990) DNA and chromosome evolution in lacertid lizards, Pages 181-204 in E. Olmo, ed. Cytogenetics of Amphibians and Reptiles. Birkhauser Verlag, Berlin, Germany.

Olmo, E., O. Cobror, A. Morescalchi, and G. Odierna. (1984) Homomorphic sex-chromosomes in the lacertid lizard Takydromus sexlineatus. Heredity 53: 457-459.

Olmo, E., Odierna, G. & Capriglione, T. (1987) Evolution of sex-chromosomes in lacertid lizards. Chromosoma 96: 33-38.

Ota, H., T. Hikida, J. Nabhitabhata, and S. Panha. 2001. Cryptic taxonomic diversity in two broadly distributed lizards of Thailand (Mabuya macularia and Dixonius siamensis) as revealed by chromosomal investigations (Reptilia: Lacertilia). Natural History Journal of Chulalongkorn University 1:1-7.

Ota, H., T. Hikida, M. Matsui, and A. Mori. 1992. Karyotypes of two species of the genus Cyrtodactylus (Squamata: Gekkonidae) from Sarawak, Malaysia. Caryologia 45:43-49.

Paez V.P., Correa J.C., Cano A.M., Bock B.C. (2009) A Comparison of Maternal and Temperature Effects on Sex, Size, and Growth of Hatchlings of the Magdalena River Turtle (Podocnemis lewyana

Peccinini D. et al. (1971) ex Olmo E., Signorino G.G. (2005) ChromoRep: A reptiles chromosomes database.

Pellegrino K.C.M. et al. (1999) ex Olmo E., Signorino G.G. (2005) ChromoRep: A reptiles chromosomes database.

Pennock L.A. et al. (1969) ex Olmo E., Signorino G.G. (2005) ChromoRep: A reptiles chromosomes database.

Pieau C. (1971) Sur la proportion sexuelle chez les embryons de deux Cheloniens (Testudo graeca L. et Emys orbicularis L.

Pieau C. (1974) Sex differentiation according to the temperature in embryos of Emys orbicularis chelonia effect of sex hormones. Annales d'Embryologie et de Morphogenese 7: 365-394.

Pokorná, M. J., M. Rovatsos, and L. Kratochvíl. 2014. Sex Chromosomes and Karyotype of the (Nearly) Mythical Creature, the Gila Monster, <italic>Heloderma suspectum</italic> (Squamata: Helodermatidae). PLoS ONE 9:e104716.

Pokorná, M., M. Rabova, P. Rab, M. A. Ferguson-Smith, W. Rens, and L. Kratochvíl. 2010. Differentiation of sex chromosomes and karyotypic evolution in the eye-lid geckos (Squamata: Gekkota: Eublepharidae), a group with different modes of sex determination. Chrom. Res. 18:809-820.

Pokorna, M., W. Rens, M. Rovatsos, and L. Kratochvil. (2014) A ZZ/ZW Sex Chromosome System in the Thick-Tailed Gecko (Underwoodisaurus milii; Squamata: Gekkota: Carphodactylidae), a Member of the Ancient Gecko Lineage. Cytogenet. Genome Res. 142:190-196.

Ray-Chaudhuri and Singh (1972) ex Olmo E., Signorino G.G. (2005) ChromoRep: A reptiles chromosomes database.

Ray-Chaudhuri S.P. et al. (1971) ex Olmo E., Signorino G.G. (2005) ChromoRep: A reptiles chromosomes database.

Ray-Chaudhuri S.P. et al. (1972) ex Olmo E., Signorino G.G. (2005) ChromoRep: A reptiles chromosomes database.

Reed K.M. et al. (1990) Synaptonemal complex-analysis of sex-chromosomes in 2 species of Sceloporus. Copeia (1990): 1122-1129.

Reed, K.M., P.D. Sudman, J.W. Sites, and I.F. Greenbaum. (1990) Synaptonemal complex-analysis of sex-chromosomes in 2 species of Sceloporus. Copeia 1990: 1122-1129.

Rimblot F., Fretey J., Mrosovsky N., Lescure J., Pieau C. (1985) Sexual differentiation as a function of the incubation temperature of eggs in the sea-turtle Dermochelys coriacea (Vandelli, 1761

Rodrigues M.T. et al. (1988) ex Olmo E., Signorino G.G. (2005) ChromoRep: A reptiles chromosomes database.

Rojo, V. et al. (2012) Sex chromosome evolution in the lizard Iberolacerta monticola (Boulenger, 1905): karyological characterization of endemic Spanish populations. Chromosome Research 20: 811-811.

Rossman D.A., Eberle W.G. (1977) Partition of the Genus Natrix, with preliminary observations on evolutionary trends in Natricine snakes. Herpetologica 33: 34-43.

Rovatsos, M., M. Altmanova, M. Pokorna, and L. Kratochvil. (2014) Conserved sex chromosomes across adaptively radiated Anolis lizards. Evolution 68:2079-2085.

Rovatsos, M., M. Pokorná, M. Altmanová, and L. Kratochvíl. (2014) Cretaceous park of sex determination: sex chromosomes are conserved across iguanas.

Saint Girons H. (1977) Caryotypes et evolution des viperes Europeennes (Reptilia, Viperidae). Bulletin De La Societe Zoologique De France 102: 39-49.

Schmid, M., C. Steinlein, T. Haaf, and A. Mijares-Urrutia. 2014. Nascent ZW Sex Chromosomes in Thecadactylus rapicauda (Reptilia, Squamata, Phyllodactylidae). Cytogenet. Genome Res. 143:259-267.

Sharma & Nakhasi (1979) ex Olmo E., Signorino G.G. (2005) ChromoRep: A reptiles chromosomes database.

Sharma, G.P. et al. (1975) Female heterogamety in the Indian cryptodiran chelonian, Kachuga smithi Gray, Pages 359-368 in K. K. Tiwari, and C. B. Srivistava, eds. Dr. B.S. Chuahah Commemoration Volume. Orissa, India, Zoological Society of India.

Shaver D.J. et al. (1988) Styrofoam box and beach temperatures in relation to incubation and sex ratios of Kemp's ridley sea turtles. In: Proceedings of the 8th Annual Workshop on Sea Turtle Conservation and Biology (ed. Schroeder BAc

Shibaike Y. et al. (2009). Chromosome Evolution in the Lizard Genus Gekko (Gekkonidae, Squamata, Reptilia) in the East Asian Islands. Cytogenetic and Genome Research 127: 182-190.

Shine & Bull (1977) ex Olmo E., Signorino G.G. (2005) ChromoRep: A reptiles chromosomes database.

Shine, R., E.G. Barrott, and M.J. Elphick. (2002) Some like it hot: Effects of forest clearing on nest temperatures of montane reptiles. Ecology 83: 2808-2815.

Singh L. et al. (1968) ex Olmo E., Signorino G.G. (2005) ChromoRep: A reptiles chromosomes database.

Singh L. et al. (1970) ex Olmo E., Signorino G.G. (2005) ChromoRep: A reptiles chromosomes database.

Singh L. et al. (1972) ex Olmo E., Signorino G.G. (2005) ChromoRep: A reptiles chromosomes database.

Singh L. et al. (1974) ex Olmo E., Signorino G.G. (2005) ChromoRep: A reptiles chromosomes database.

Singh L. et al. (1979) ex Olmo E., Signorino G.G. (2005) ChromoRep: A reptiles chromosomes database.

Singh L. et al. (1980) ex Olmo E., Signorino G.G. (2005) ChromoRep: A reptiles chromosomes database.

Singh, L. (1972) Evolution of karyotypes in snakes. Chromosoma 38: 185-236.

Solorzano A. et al. (1989) ex Olmo E. (2005) Rate of chromosome changes and speciation in reptiles. Genetica 125: 185-203.

Spotila J.R. et al. (1994) Effects of incubation conditions on sex determination, hatching success, and growth of hatchling desert tortoises, Gopherus agassizii. Herpetol. Monog. 8: 103-116.

Stewart, S., D. Morafka, and A. Stock. (1990) Karyotypes of Gulf of California insular rattlesnakes (Viperidae: Crotalus) compared to those of peninsular sister taxa: Cytogenetics of amphibians and reptiles.

Sulandari, S., M. S. A. Zein, E. A. Arida, and A. Hamidy. (2014) Molecular sex determination of captive Komodo dragons (Varanus komodoensis) at Gembira Loka zoo, Surabaya zoo, and Ragunan zoo, Indonesia. Hayati Journal of Biosciences 21:65-75.

Tang, X.-L., F. Yue, X.-F. Yan, D.-J. Zhang, Y. Xin, C. Wang, and Q. Chen. (2012) Effects of gestation temperature on offspring sex and maternal reproduction in a viviparous lizard (Eremias multiocellata) living at high altitude. Journal of Thermal Biology 37: 438-444.

Telemeco RS. (2015) Sex Determination in Southern Alligator Lizards (Elgaria multicarinata; Anguidae). Herpetologica 71: 8-11. Weak data (too small N); Harlow 2004. (Gerrhonotus) listed as weakly supported TSD.

Toriba M. (1990) ex Olmo E., Signorino G.G. (2005) ChromoRep: A reptiles chromosomes database.

Trifonov, V. A., M. Giovannotti, P. C. M. O'Brien, M. Wallduck, F. Lovell, W. Rens, P. P. Parise-Maltempi, V. Caputo, and M. A. Ferguson-Smith. 2011. Chromosomal evolution in Gekkonidae. I. Chromosome painting between Gekko and Hemidactylus species reveals phylogenetic relationships within the group. Chrom. Res. 19:843-855.

Uller T, Mott B, Odierna G, Olsson M. (2006) Consistent sex ratio bias of individual female dragon lizards. Biol Lett 2: 569-572.

Valenzuela et al. 2015, submitted

Valenzuela N. (2001) Constant, shift and natural temperature effects on sex determination in Podocnemis expansa turtles. Ecology 82: 3010-3024.

Valenzuela, N. (2004) Temperature-dependent sex determination. In: Reptilian Incubation: Environment & Behaviour, (Deeming, D. C., ed.). pp. 211-227. Nottingham University Press, Nottingham, UK.

Valenzuela N., Adams D.C., Janzen F.J. (2003) Pattern does not equal process: exactly when is sex environmentally determined? Am. Nat. 161: 676-683.

Viets B.E., Ewert M.A., Talent L.G., Nelson C.E. (1994) Sex-determining mechanisms in squamate reptiles. The Journal of Experimental Zoology 270: 45-56.

Vogt R.C. (2001) in Berhard 2010 PhD Thesis.

Vogt R.C. (2008) Amazon turtles. Grafica Biblos, Lima, Peru.

Vogt R.C., Flores-Villela O. (1992) Effects of incubation temperature on sex determination in a community of neotropical fresh-water turtles in southern Mexico. Herpetologica 48: 265-270.

Volobouev, V., Pasteur G. (1988) Presumptive sex chromosomes of a unisexual homomorphic species of lizards, Lepidodactylus lugubris. Heredity 60: 463-467.

Wapstra E. et al. (2004) Maternal basking behaviour determines offspring sex in a viviparous reptile. Proceedings of the Royal Society of London. Series B: Biological Sciences 271: S230-S232.

Webb G.J., Choquenot D., Whitehead P.J. (1986) Nests, eggs, and embryonic development of Carettochelys insculpta (Chelonia: Carettochelidae

Yntema C.L. (1976) Effects of incubation temperature on sexual differentiation in the turtle, Chelydra serpentina . Journal of Morphology 150: 453-462.

Yntema C.L., Mrosovsky N. (1979) Incubation Temperature and Sex Ratio in Hatchling Loggerhead Turtles: A Preliminary Report. Marine Turtle Newsletter 11: 9-10.

Yonenaga-Yassuda & Rodrigues (1999) ex Olmo E., Signorino G.G. (2005) ChromoRep: A reptiles chromosomes database.

Yonenaga-Yassuda Y. et al. (1998) ex Olmo E., Signorino G.G. (2005) ChromoRep: A reptiles chromosomes database.

Yoshida and Msahiro (1974) ex Moritz C. (1990) Patterns and process of sex chromosome evolution in gekkonid lizards (Sauria: Reptilia), Pages 205-219, Cytogenetics of Amphibians and Reptiles, Birkhauser Verlag Basel.

Zeng, X. M., Y. Z. Wang, Z. J. Liu, Z. L. Fang, and G. F. Wu. (1997) Karyotypes of nine species in the geneus Phrynocephalus, with discussion of karyotypic evolution of Chinese Phrynocephalus. Acta Zoologica Sinica: 399-410.

Zhang D-J, Tang X-L, Yue F, et al. (2010) Effect of gestation temperature on sexual and morphological phenotypes of offspring in a viviparous lizard, Eremias multiocellata. Journal of Thermal Biology 35: 129-133.

Table S1b: Taxonomic coverage of turtle and squamate families used in this study. Family names follow Uetz and Hosek (2015).

Order	Vernacular	Family	Number Species	Species known SDM	% Species known SDM
Squamata	lizards	Agamidae	466	26	5.6
Squamata	lizards	Anguidae	75	1	1.3
Squamata	lizards	Anniellidae	6	0	0
Squamata	lizards	Carphodactylidae	30	1	3.3
Squamata	lizards	Chamaeleonidae	202	4	2.0
Squamata	lizards	Cordylidae	67	0	0
Squamata	lizards	Corytophanidae	9	2	22.2
Squamata	lizards	Crotaphytidae	12	2	16.7
Squamata	lizards	Dactyloidae	400	51	12.8
Squamata	lizards	Dibamidae	23	1	4.3
Squamata	lizards	Diplodactylidae	137	6	4.4
Squamata	lizards	Diploglossidae	51	0	0
Squamata	lizards	Eublepharidae	36	9	25.0
Squamata	lizards	Gekkonidae	1063	34	3.2
Squamata	lizards	Gerrhosauridae	37	0	0
Squamata	lizards	Gymnophthalmidae	253	7	2.8
Squamata	lizards	Helodermatidae	2	1	50.0
Squamata	lizards	Hoplocercidae	19	0	0
Squamata	lizards	Iguanidae	41	3	7.3
Squamata	lizards	Lacertidae	322	47	14.6
Squamata	lizards	Lanthanotidae	1	0	0
Squamata	lizards	Leiocephalidae	29	1	3.4
Squamata	lizards	Leiosauridae	33	1	3.0
Squamata	lizards	Liolaemidae	298	1	0.3
Squamata	lizards	Opluridae	8	2	25.0
Squamata	lizards	Phrynosomatidae	148	48	32.4
Squamata	lizards	Phyllodactylidae	134	8	6.0
Squamata	lizards	Polychrotidae	7	3	42.9
Squamata	lizards	Pygopodidae	46	3	6.5
Squamata	lizards	Scincidae	1602	19	1.2
Squamata	lizards	Shinisauridae	1	0	0
Squamata	lizards	Sphaerodactylidae	215	4	1.9
Squamata	lizards	Teiidae	151	3	2.0
Squamata	lizards	Tropiduridae	128	6	4.7
Squamata	lizards	Varanidae	78	9	11.5
Squamata	lizards	Xantusiidae	34	0	0
Squamata	lizards	Xenosauridae	10	0	0
Squamata	snakes	Acrochordidae	3	0	0
Squamata	snakes	Aniliidae	1	0	0
Squamata	snakes	Anomalepididae	18	0	0
Squamata	snakes	Anomochilidae	3	0	0
Squamata	snakes	Boidae	59	3	5.1
Squamata	snakes	Bolyeridae	2	0	0
Squamata	snakes	Colubridae	851	23	2.7
Squamata	snakes	Cylindrophiidae	13	0	0
Squamata	snakes	Dipsadidae	754	18	2.4
Squamata	snakes	Elapidae	289	74	25.6
Squamata	snakes	Elapidae	71	0	0
Squamata	snakes	Gerrhopilidae	18	0	0
Squamata	snakes	Homalopsidae	53	2	3.8
Squamata	snakes	Lamprophiidae	308	0	0
Squamata	snakes	Leptotyphlopidae	126	0	0
Squamata	snakes	Loxocemidae	1	0	0
Squamata	snakes	Natricidae	226	14	6.2
Squamata	snakes	Pareatidae	20	0	0
Squamata	snakes	Pseudoxenodontidae	10	0	0
Squamata	snakes	Pythonidae	40	4	10.0
Squamata	snakes	Tropidophiidae	34	0	0
Squamata	snakes	Typhlopidae	263	0	0
Squamata	snakes	Uropeltidae	54	0	0
Squamata	snakes	Viperidae	331	36	10.9
Squamata	snakes	Xenodermatidae	18	0	0
Squamata	snakes	Xenopeltidae	2	0	0
Squamata	snakes	Xenophidiidae	2	0	0
Squamata	snakes	Xenotyphlopidae	1	0	0
Chelonia	turtles	Carettochelyidae	1	1	100
Chelonia	turtles	Chelidae	58	9	15.5
Chelonia	turtles	Cheloniidae	6	6	100
Chelonia	turtles	Chelydridae	5	2	40.0
Chelonia	turtles	Dermatemydidae	1	1	100
Chelonia	turtles	Dermochelyidae	1	1	100
Chelonia	turtles	Emydidae	52	23	44.2
Chelonia	turtles	Geoemydidae	69	11	15.9
Chelonia	turtles	Kinosternidae	25	17	68.0
Chelonia	turtles	Pelomedusidae	27	2	7.4
Chelonia	turtles	Platysternidae	1	0	0
Chelonia	turtles	Podocnemididae	8	5	62.5
Chelonia	turtles	Testudinidae	58	6	10.3
Chelonia	turtles	Trionychidae	32	3	9.4

2. Figure S1

Ml ancestral reconstruction of sex-determining mechanisms in (A) squamates, and using the alternative SDM classification in (B) squamates and (C) lizards.

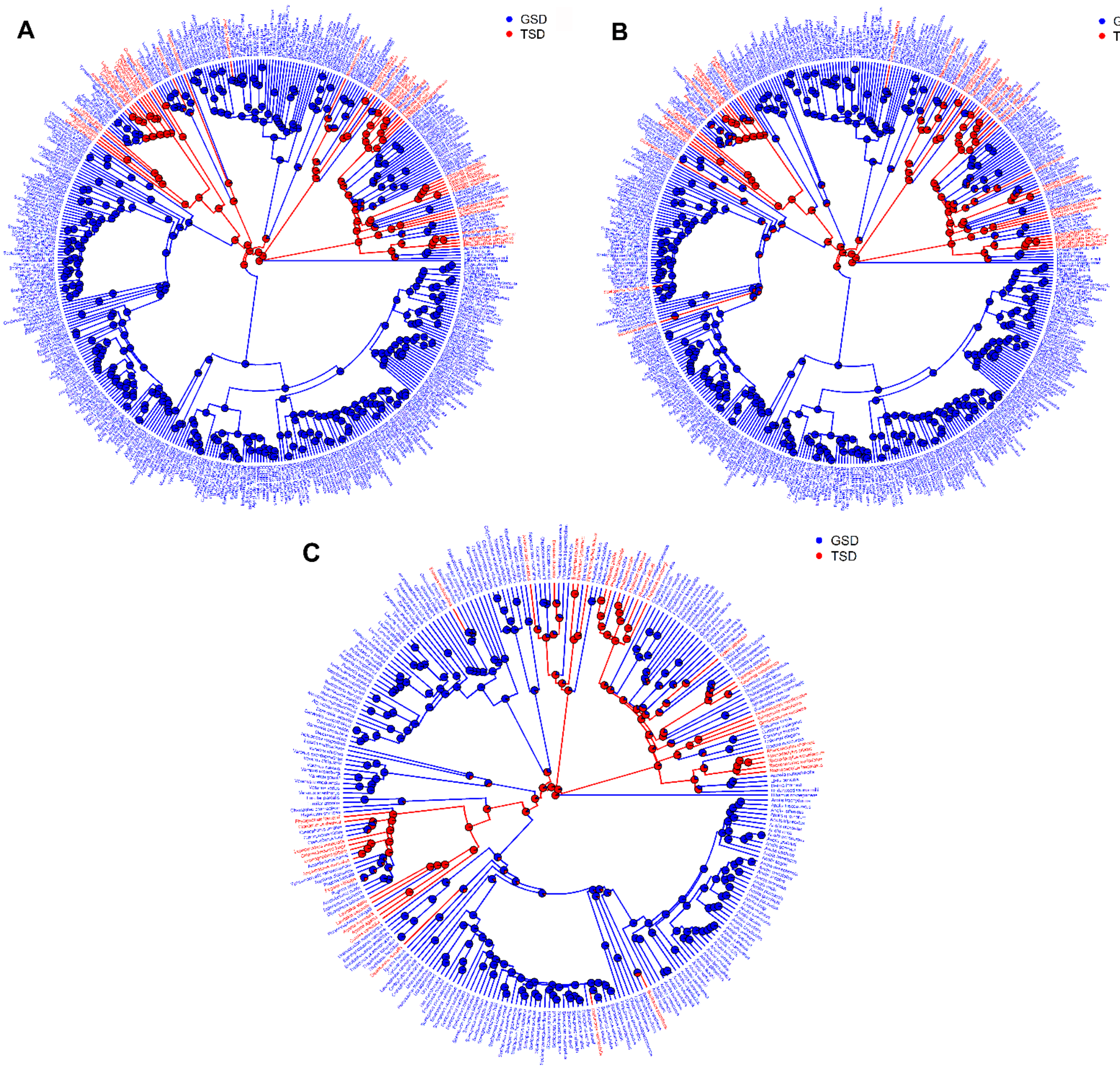

3. Results using alternative SDM assignment for species with mixed or equivocal SDM as listed in Table S1

Table S2: MacroCAIC results using alternative SDM assignment

Group	MNS [a]	r^2	Slope	*p* value	Simulation *p* value
Turtles	10	-0.05	3.56	0.717	0.76
	20	0.06	33.30	0.276	0.34
	30	0.71	66.90	0.046	0.17
	40	0.71	66.90	0.046	0.19
Lizards	10	-0.01	-1.42	0.858	0.77
	20	-0.02	2.91	0.879	0.88
	30	-0.02	-10.04	0.683	0.62
	40	-0.03	-14.05	0.592	0.60
Squamates	10	-0.01	-1.63	0.832	0.84
	20	-0.02	1.97	0.913	0.90
	30	-0.02	-11.21	0.634	0.55
	40	-0.02	-15.22	0.538	0.44

[a] MNS: minimal number of species included for computing contrasts.

Table S3: BAMM estimation for the number of rate shifts in diversification. The number of rates shifts with the highest probability in each group is marked in bold.

Group	Number of Shifts	Probability
Turtles	**1**	**0.81**
	2	0.18
	3	0.02
	4	0.00
Lizards	1	0.05
	2	0.08
	3	0.09
	4	0.13
	5	0.06
	6	0.08
	**7**	**0.19**
	8	0.15
	9	0.09
	10	0.04
	11	0.02
	12	0.01
	13	0.00
	14	0.00
Squamates	2	0.04
	3	0.05
	4	0.03
	5	0.03
	6	0.04
	7	0.05
	8	0.07
	9	0.08
	10	0.08
	11	0.06
	12	0.04
	13	0.04
	14	0.05
	15	0.11
	**16**	**0.12**
	17	0.08
	18	0.03
	19	0.01
	20	0.00
	21	0.00

Table S4: Summary of transition rate parameters estimates using the MK2 model with both Maximum Likelihood and Bayesian (MCMC) methodologies and BiSSE for the turtles, lizards, and squamate data sets using the alternative SDM assignment.

Group	Analysis	q_{GT}	q_{TG}	Significance [a]	Simulation *p* value
**Turtles**					
	Maximum Likelihood	8.6e-07	0.0017	0.10	0.15
	MCMC	0.0015	0.0022	0.76	0.06
	BiSSE	6.5e-06	0.0018	0.10	0.14
**Lizards**					
	Maximum Likelihood	0.0015	0.0177	**6.6e-06**	**<0.001**
	MCMC	2.0e-03	0.0180	**1**	**<0.001**
	BiSSE	0.0015	0.0177	6.6e-06	**<0.001**
**Squamates**					
	Maximum Likelihood	7.0e-04	0.0177	**3.2e-09**	**<0.001**
	MCMC	9.5e-04	0.0181	**1**	**0.001**
	BiSSE	7.0e-04	0.0177	3.2e-08	**<0.001**

[a] Significance is estimated with Likelihood-ratio-test for the MK2 and BiSSE Maximum Likelihood analyses; Significance of the MCMC analyses is estimated by calculating the proportion of MCMC steps (i.e., the posterior probability, *PP)* in which q_{TG} was higher than q_{GT}. *PP* value above 0.975 or below 0.025 indicates a significant difference between the two rates.

Table S5: Log likelihood differences (ΔLL) obtained between the single (BM1) and two rate (BM2) Brownian motion models of evolution, and between the single (OU1) and two (OU2) optimums, as estimated for lifepan in turtles, lizards and squamates. σ^2_{GSD} and σ^2_{TSD}, Optimum$_{GSD}$, and Optimum$_{TSD}$: estimated parameters for GSD and TSD lineages using the alternative SDM assignment.

Group	LogLiks BM1	LogLiks BM2	BM p-value[a]	σ2 GSD	σ2 TSD	LogLiks OU1	LogLiks OU2	OU p-value[b]	Optimum GSD	Optimum TSD
Turtles	-90.7	-84.7	**0.0005**	0.36	2.84	-72.9	-70.1	**0.0181**	22.6	35.9
Lizards	-192.9	-190.3	**0.0234**	3.21	1.71	-163.8	-162.4	0.0928	7.8	10.6
Squamates	-246.4	-245.3	0.1270	2.53	1.71	-220.7	-220.7	0.7048	9.4	10.2

[a]*p*-value comparing the fit of a single and two rate BM models based on the likelihood ratio test.
[b]*p*-value comparing the fit of a single and two OU models based on the likelihood ratio test. Significant *p*-values are marked in bold.

4. BiSSE ANALYSES

GENERAL METHODS

We applied the BiSSE framework (Maddison et al., 2007) as implemented in *diversitree* version 0.9.7 (FitzJohn, 2012), using the "skeletal" tree approach (FitzJohn et al., 2009) which accounts for the sampling fraction of species in the phylogeny out of the total number of species in the clade (assuming an equal sampling fraction for both TSD and GSD). This method was used to estimate the speciation rates of lineages in states GSD and TSD (λ_G and λ_T, respectively), extinction rates (μ_G and μ_T) and transition rates from GSD to TSD ($q_{G\rightarrow T}$) and from TSD to GSD ($q_{T\rightarrow G}$). The net diversification rate in each state (r_G and r_T), was calculated as $r_G = \lambda_G - \mu_G$ and $r_T = \lambda_T - \mu_T$.

We used maximum likelihood (ML) to test whether GSD and TSD lineages (1) speciate at different rates, (2) go extinct at different rates, and (3) whether the transition rate from GSD to TSD is different than from TSD to GSD. These three, non-mutually-exclusive, hypotheses were tested by comparing the following BiSSE models, starting with the null model, M0, in which $\lambda_G = \lambda_T$, $\mu_G = \mu_T$, and $q_{G\rightarrow T} = q_{T\rightarrow G}$, up to the most general model in which all rate parameters are allowed to differ between GSD and TSD (**Table S6**). To increase the probability of finding the global optimum, we started the ML search from 100 different points uniformly sampled along the range [0,1] of each of the model rate parameters. The Akaike information criterion (AIC) was used to choose between the competing models (Arnold, 2010).

Table S6: Summary of all models used in the ML analysis. Models are ordered according to increased complexity.

Model (number of parameters)	Speciation	Extinction	Transition
M0 (3)	$\lambda_G = \lambda_T$	$\mu_G = \mu_T$	$q_{G\rightarrow T} = q_{T\rightarrow G}$
Ms (4)	$\lambda_G \neq \lambda_T$	$\mu_G = \mu_T$	$q_{G\rightarrow T} = q_{T\rightarrow G}$
Me (4)	$\lambda_G = \lambda_T$	$\mu_G \neq \mu_T$	$q_{G\rightarrow T} = q_{T\rightarrow G}$
Mq (4)	$\lambda_G = \lambda_T$	$\mu_G = \mu_T$	$q_{G\rightarrow T} \neq q_{T\rightarrow G}$
Mse (5)	$\lambda_G \neq \lambda_T$	$\mu_G \neq \mu_T$	$q_{G\rightarrow T} = q_{T\rightarrow G}$
Msq (5)	$\lambda_G \neq \lambda_T$	$\mu_G = \mu_T$	$q_{G\rightarrow T} \neq q_{T\rightarrow G}$
Meq (5)	$\lambda_G = \lambda_T$	$\mu_G \neq \mu_T$	$q_{G\rightarrow T} \neq q_{T\rightarrow G}$
Mseq (6)	$\lambda_G \neq \lambda_T$	$\mu_G \neq \mu_T$	$q_{G\rightarrow T} \neq q_{T\rightarrow G}$

SIMULATION ANALYSES TO TEST FOR BiSSE SENSITIVITY

Estimation of diversification rates could be influenced by additional factors other than the trait of interest (here SDM), that can alter the tree shape in a way that elevates the false-positive rate (FitzJohn, 2012, Rabosky & Goldberg, 2015). We thus compared the log-likelihood difference (ΔLL) for the competing models as inferred using our empirical data against those obtained using data generated by simulating characters that do not influence diversification. Specifically, we used a parametric bootstrapping approach to obtain the null distribution of the competing BiSSE models (i.e., equal versus unequal speciation models). We simulated 100 random distributions of neutral characters (assuming no effect on diversification) on the same empirically-derived phylogenies of turtles and lizards. To obtain the simulated parameter values, we first estimated the two transition rates (GSD to TSD and TSD to GSD) according to a BiSSE model with equal extinction and speciation (Mq) and the root state set to TSD (which was inferred as the root state, see Results). We then simulated a binary trait along the tree [using sim.character function within the package *diversitree* (FitzJohn, 2012)] with the transition rates estimated using the MK2 model (with the root state set to TSD). We then applied BiSSE to compare between a model of unequal speciation (denoted Msq for unequal speciation and transition rates) with a nested model that assumes equal speciation rates but unequal transition rates (denoted Mq for unequal transition rates). In both models, extinction is modeled as equal because extinction rates are difficult to estimate and are particularly sensitive to sampling biases (see Results). This procedure resulted in an expected distribution of ΔLL under the null model. Finally, the empirically-derived ΔLL between models Msq and Mq in the real data was compared to the corresponding simulated distributions to obtain a *p* value according to the proportion of simulated ΔLL values in the simulated data that were equal or greater than the observed value. We applied BiSSE twice, first with the complete trees, to make use of as much phylogenetic data as possible, and second, after the trees were pruned to include only taxa with known SDM, so that the results could be compared to the results of the rest of the analyses.

RESULTS

We first used the BiSSE approach (Maddison et al., 2007) to test whether SDM is associated with altered diversification rates in turtles and lizards. The Ms (unequal speciation) was identified as the best-fitted model for **turtles** (ΔAIC = 0), suggesting that speciation rates were higher for TSD than for GSD lineages, whereas extinction rates estimates were near zero and indistinguishable between SDMs (**Table S7**). We note that extinction rates are difficult to estimate and are particularly sensitive to sampling biases [(Rabosky, 2010); but see (Beaulieu & O'Meara, 2015)].

In lizards, Msq (unequal speciation and transition) was the best model-fitted, suggesting that speciation rates were higher for GSD than for TSD lineages but that transition rate from TSD to GSD was significantly higher than the transition rate from GSD to TSD.

In squamates, Mseq (unequal speciation, extinction, and transition) was the best model-fitted, suggesting that speciation and extinction rates were higher for GSD than for TSD lineages but that transition rate from TSD to GSD was significantly higher than the transition rate from GSD to TSD. Most importantly however, results from our parametric bootstrapping procedure using neutral binary traits, showed that in all groups, at least 65% of the simulations resulted in ΔLL values that are equal or greater than the observed value. Thus, the observed differences in rates inferred using BiSSE are not significantly different than what can be expected by chance. When pruned trees were used, some of the estimated rates and chosen models were different. However, similar to the results obtained with the full trees, the parametric bootstrapping showed that in all groups, 42-58% of the simulations resulted in ΔLL values that are equal or greater than the observed value, suggesting, again, that the observed differences in rates are not significantly different than what can be expected by chance.

Table S7: Summary of parameters estimates using the best-fitted BiSSE model for the turtles, lizards, and squamates data sets using the full phylogenies. The Ms model (ΔAIC=0) was chosen in turtles, whereas Msq was chosen in lizards. λ= Speciation rate; μ = extinction rate; $q_{G\to T}$ = transition rate from GSD to TSD; $q_{T\to G}$ = transition rate from TSD to GSD. ΔAIC is the difference in AIC of each model relative to the best supported model. Parameter estimates are given based on the best supported model for each dataset.

Group	Parameter estimates						ΔAIC							
	λ_G	λ_T	μ_G	μ_T	$q_{G\to T}$	$q_{T\to G}$	M0	Ms	Me	Mq	Mse	Msq	Meq	Mseq
**Turtles**	0.0223	0.0463	0	0	0.0018	0.0018	23.8	0.0	23.1	23.1	2.0	2.0	25.1	4.0
**Lizards**	0.0319	0.1384	0	0	0.0063	0.1096	559.8	547.2	532.0	544.1	549.2	0.0	546.1	2.0
**Squamates**	0.0381	0.1691	0	0	0.0049	0.1327	960.3	1028.3	776.5	935.8	1002.2	0.0	776.8	2.0

Results using alternative SDM assignment for species with mixed or equivocal SDM (see text for details)

Group	Parameter estimates						ΔAIC							
	λ_G	λ_T	μ_G	μ_T	$q_{G\to T}$	$q_{T\to G}$	M0	Ms	Me	Mq	Mse	Msq	Meq	Mseq
**Lizards**	0.032	0.1415	0	0	0.007	0.1162	601.2	553.2	551.8	583.0	555.2	0.0	554.3	2.0
**Squamates**	0.0383	0.1724	0	0	0.0052	0.139	1010.2	1037.7	799.5	981.6	1011.5	0.0	800.3	2.0

Table S8: Summary of parameters estimates using the best-fitted BiSSE model for the turtles, lizards, and squamates data sets using the pruned phylogenies that contain only data with SDM information. The Ms model (ΔAIC=0) was chosen in turtles, whereas Msq was chosen in lizards. λ= Speciation rate; μ = extinction rate; $q_{G\to T}$ = transition rate from GSD to TSD; $q_{T\to G}$ = transition rate from TSD to GSD. ΔAIC is the difference in AIC of each model relative to the best supported model. Parameter estimates are given based on the best supported model for each dataset.

Group	Parameter estimates						ΔAIC							
	λ_G	λ_T	μ_G	μ_T	$q_{G\to T}$	$q_{T\to G}$	M0	Ms	Me	Mq	Mse	Msq	Meq	Mseq
**Turtles**	0.0273	0.0563	0.0101	0.0101	0.0019	0.0019	10	0	1.83	9.25	1.89	1.96	3.03	3.88
**Lizards**	0.3737	0.3584	0.3286	0.3286	7.00E-04	0.0089	16.92	13.31	16.04	1.21	12.5	0	0.21	1.01
**Squamates**	0.4967	0.2449	0.4431	0.2088	3.00E-04	0.0124	37.32	21.52	20.64	12.89	21.77	1.11	1.77	0

Results using alternative SDM assignment for species with mixed or equivocal SDM (see text for details)

Group	Parameter estimates						ΔAIC							
	λ_G	λ_T	μ_G	μ_T	$q_{G\to T}$	$q_{T\to G}$	M0	Ms	Me	Mq	Mse	Msq	Meq	Mseq
**Lizards**	0.4378	0.2131	0.3986	0.1621	0.0011	0.0269	18.44	4.22	4.64	0.14	5.92	1.52	1.77	0
**Squamates**	0.4523	0.0796	0.3837	0.0205	7.00E-04	0.028	49.65	24.55	25.7	21.06	26.2	12.73	14.42	0

DIVERSIFICATION ANALYSES USING MARKOV CHAIN MONTE CARLO (MCMC) SAMPLING

A Markov chain Monte Carlo (MCMC) sampling approach described in (FitzJohn et al., 2009) was used to estimate the posterior probability distributions for each of the six parameters. Posterior distributions were estimated using an exponential prior distribution (with mean set to twice the maximal ML rate estimate under a trait-independent model; $\lambda_G = \lambda_T$, $\mu_G = \mu_T$, $q_{G\to T} = q_{G\to T}$) placed on the six parameters. MCMC chains were started at the estimated parameters through ML, and were run for 10,000 steps; the first 10% of the steps were discarded as burn-in.

To test whether estimated extinction and speciation rates differ between TSD and GSD lineages, we calculated the percentage of BiSSE MCMC steps in which the GSD rate was higher than that of the TSD state (i.e., the posterior probability, *PP*, of GSD lineages having a higher rate than TSD lineages). For example, to test whether extinction rates differ, we calculated the percentage of post burn-in steps in which $\mu_G > \mu_T$, and interpreted $PP(\mu_G > \mu_T) \geq 0.975$ as significant support for the conclusion that GSD lineages go extinct at a higher rate than TSD ones, with the converse, $PP(\mu_G > \mu_T) \leq 0.025$, supporting higher TSD extinction.

To ensure that the MCMC search sample throughout the parameter space, we ran two chains starting from the top two MLE points. In the squamates/lizard datasets these two chains failed to converge, getting stuck in separate hills of the likelihood surface, leading to opposite interpretation of the data (Figure S1). The chain that resulted in higher TSD speciation (Figure S1a), which is compatible with the ML analysis presented in the main text, sampled the parameter space at substantially higher likelihood surface compared to the chain that resulted in higher GSD speciation (Figure S1b) (average difference between the two chain ca. 80 log-likelihood values).

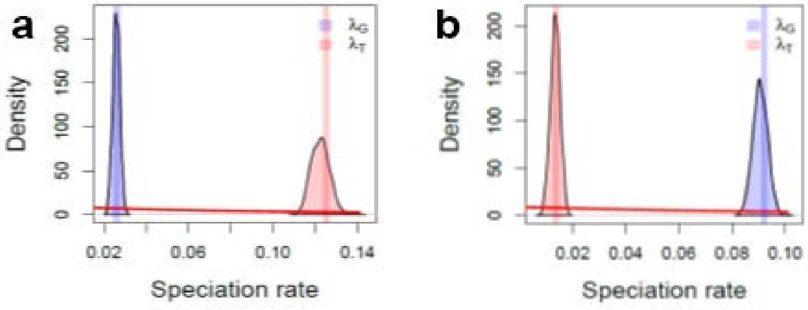

Figure S2. BiSSE MCMC results of using the two best MLE points as the starting points for the MCMC sampler using the lizard dataset. Posterior probability density distributions from MCMC analyses are shown for speciation rates for GSD (pink) and TSD lineages (blue). The MCMC starting point values of each parameter are marked by vertical lines. The red lines show the prior distribution (set according to a trait-independent model). (a) MCMC chain was initiated from the best-fitted ML point, leading to estimation of higher speciation rate in TSD lineages. (b) MCMC chain was initiated from the second-best-fitted ML point, leading to estimation of higher speciation rate in GSD lineages.

SIMULATIONS TO TEST EFFECT OF MISSING DATA ON TRANSITION RATE ESTIMATES IN BiSSE

We also tested the effect of missing data on the estimation of the transition rates in BiSSE, given that information for a substantial portion of extant lizards is lacking. For this, we simulated random trees with 1,000 tips with equal speciation rates (lambda = 0.1), no extinction, and varying transition rates (q01 = 0.1, q10 = 0.1, 0.05, 0.025) and carried out 100 simulations for each parameter combination. In each simulation, the data were analyzed by BiSSE with 100, 25, or 5% of the state data (Figure S3). The estimated transition rates are shown in Figure S3. The results illustrate that missing data leads to increased variance in the estimated transition rates, although the average estimation is rather accurate.

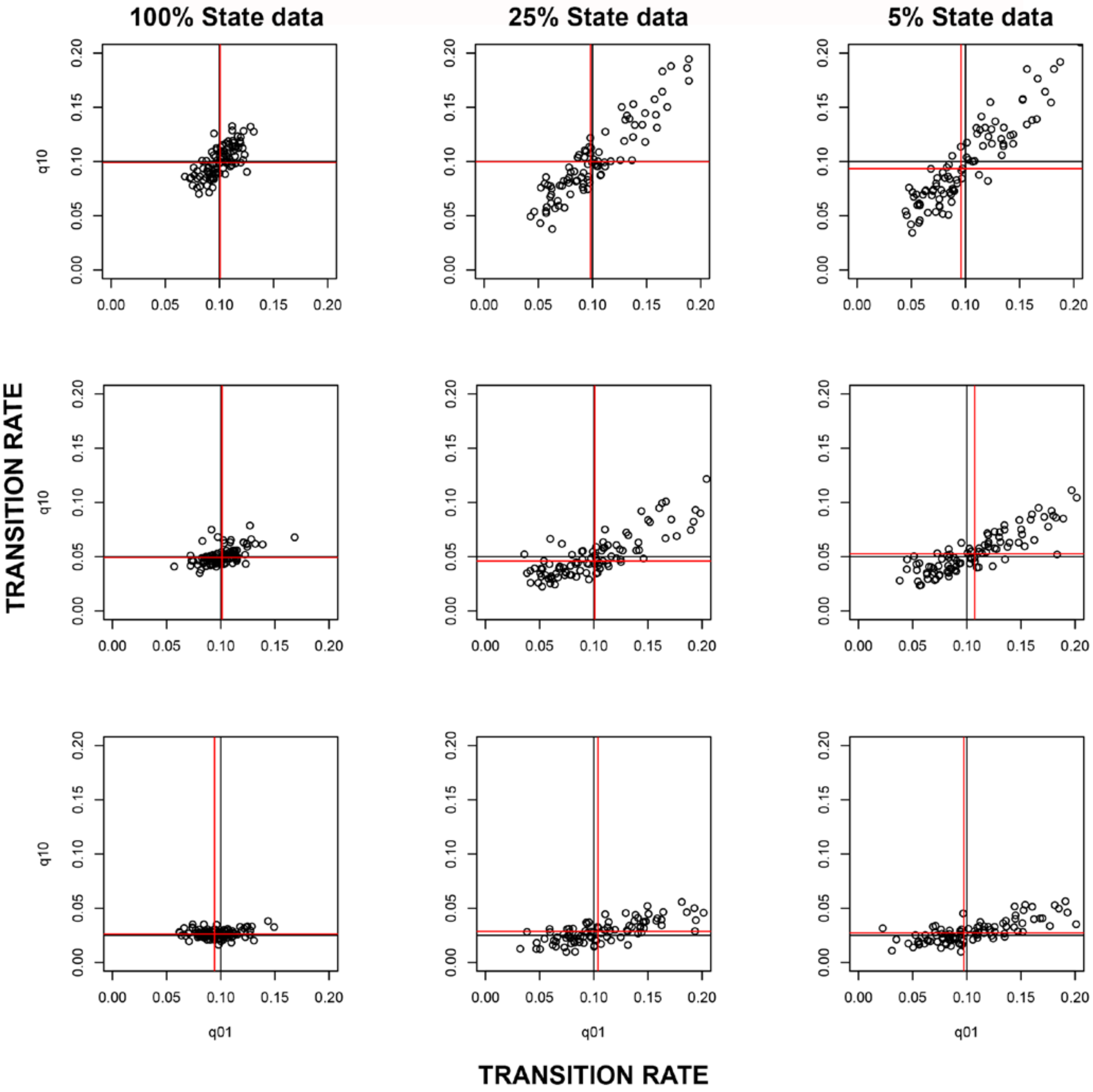

Figure S3. Effect of missing data on the estimation of transition rates in BiSSE. Columns correspond to the three level of data completeness (left: 100%, middle: 25%, and right: 5%) and rows correspond to three values of simulated q10 (top: 0.1, middle: 0.05, and bottom: 0.025). The black lines mark the simulated values and the red lines mark the median of the estimated values.

References

Arnold, T. W. 2010. Uninformative Parameters and Model Selection Using Akaike's Information Criterion. *Journal of Wildlife Management* **74**: 1175-1178.

Beaulieu, J. M. & O'Meara, B. C. 2015. Extinction can be estimated from moderately sized molecular phylogenies. *Evolution* **69**: 1036-1043.

FitzJohn, R. G. 2012. Diversitree: comparative phylogenetic analyses of diversification in R. *Methods in Ecology and Evolution* **3**: 1084-1092.

FitzJohn, R. G., Maddison, W. P. & Otto, S. P. 2009. Estimating trait-dependent speciation and extinction rates from incompletely resolved phylogenies. *Systematic Biology* **58**: 595-611.

Maddison, W. P., Midford, P. E. & Otto, S. P. 2007. Estimating a binary character's effect on speciation and extinction. *Systematic Biology* **56**: 701-710.

Rabosky, D. L. 2010. Extinction rates should not be estimated from molecular phylogenies. *Evolution* **64**: 1816-1824.

Rabosky, D. L. & Goldberg, E. E. 2015. Model inadequacy and mistaken inferences of trait-dependent speciation. *Systematic Biology* **64**: 340-55.

Uetz, P. & Hosek, J. 2015. The Reptile Database. (Accessed 20 March 2016). Available at http://www.reptile-database.org/.
